# Sexually dimorphic dynamics of the microtubule network in medaka (*Oryzias latipes*) germ cells

**DOI:** 10.1242/dev.201840

**Published:** 2024-03-13

**Authors:** Mariko Kikuchi, Miyo Yoshimoto, Tokiro Ishikawa, Yuto Kanda, Kazutoshi Mori, Toshiya Nishimura, Minoru Tanaka

**Affiliations:** ^1^ Division of Biological Science, Graduate School of Science, Nagoya University, Nagoya, Aichi 464-8602, Japan; ^2^ Department of Biophysics, Graduate School of Science, Kyoto University, Kyoto 606-8502, Japan

**Keywords:** Germline sexual differentiation, Microtubule, Oogenesis, Balbiani body, Medaka

## Abstract

Gametogenesis is the process through which germ cells differentiate into sexually dimorphic gametes, eggs and sperm. In the teleost fish medaka (*Oryzias latipes*), a germ cell-intrinsic sex determinant, *foxl3*, triggers germline feminization by activating two genetic pathways that regulate folliculogenesis and meiosis. Here, we identified a pathway involving a dome-shaped microtubule structure that may be the basis of oocyte polarity. This structure was first established in primordial germ cells in both sexes, but was maintained only during oogenesis and was destabilized in differentiating spermatogonia under the influence of Sertoli cells expressing *dmrt1*. Although *foxl3* was dispensable for this pathway, *dazl* was involved in the persistence of the microtubule dome at the time of gonocyte development. In addition, disruption of the microtubule dome caused dispersal of *bucky ball* RNA, suggesting the structure may be prerequisite for the Balbiani body. Collectively, the present findings provide mechanistic insight into the establishment of sex-specific polarity through the formation of a microtubule structure in germ cells, as well as clarifying the genetic pathways implementing oocyte-specific characteristics.

## Teaser

**Summary:** A dome-shaped microtubule structure is identified in medaka that is present prior to sex determination in primordial germ cells and is maintained in females, contributing to oocyte polarity.

## INTRODUCTION

After germ cells commit to oogenesis or spermatogenesis, sexually different genetic pathways must be activated to direct the development of functional oocytes or sperm. During oogenesis, germ cells halve their genome through meiosis while developing within follicles to increase cell size and accumulate maternal factors necessary for embryogenesis. In addition, oocytes acquire polarity and form an animal–vegetal axis that leads to the embryonic body axis in non-mammalian vertebrates (Fujisue et al., 1993; Marlow and Mullins, 2008). However, the initial pathways that are integrated and activated at the time of germ cell sex determination remain largely unknown in vertebrates.

The teleost fish medaka (*Oryzias latipes*) is an ideal vertebrate model for analyzing the mechanism of germ cell sex determination and the initial steps of gametogenesis. The sex of medaka is genetically determined by the sex-determining gene *DMY* (*dmrt1bY*) on the Y chromosome (Matsuda et al., 2002; Nanda et al., 2002). The presence of sexually indifferent germline stem cells in both ovaries and testes enables the examination of the mechanisms underlying germline sex determination (Nakamura et al., 2010; Tanaka, 2016; Kikuchi and Tanaka, 2022). Use of medaka as a model identified Foxl3 (Forkhead box L3) as a germ cell-intrinsic factor that is required for sexual fate decisions in germ cells (Nishimura et al., 2015). Foxl3 is expressed in mitotically active oogonia, including germline stem cells, and directly triggers two oogenesis pathways via upregulation of *rec8a* and *fbxo47*, which regulate meiosis progression and follicle formation, respectively (Kikuchi et al., 2019; Kikuchi et al., 2020). However, a genetic pathway mediating the establishment of oocyte polarity remains to be integrated with germ cell sex determination.

In this study, we identified a genetic pathway that regulates microtubule organization. We discovered a dome-shaped microtubule (‘MT dome’) structure that is formed in primordial germ cells (PGCs) and is maintained in stem-type germ cells in both sexes. After germ cell sex determination, the structure is destabilized in spermatogonia, persists during oogenesis, and is replaced with the Balbiani body (Bb), which forms at the zygotene stage of meiotic prophase I. Genetic analyses indicate that the structure is not under the control of *foxl3* during oogenesis, and thus represents a previously unappreciated pathway regulating the female-specific retention of the MT dome and contributing to egg polarity.

## RESULTS

### The stability of the MT dome differs between oogenesis and spermatogenesis

**Fig. 1.**
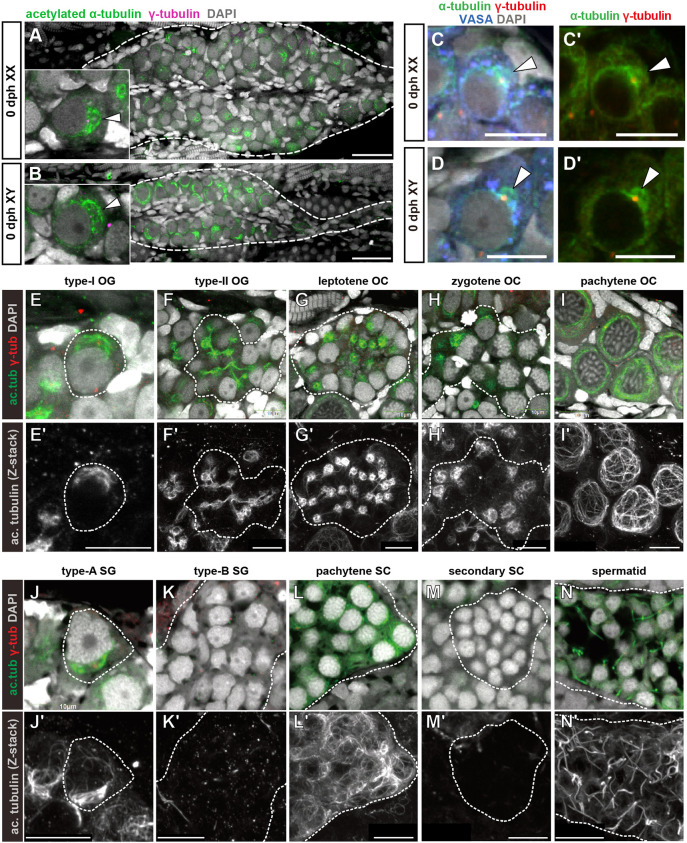
**Sexually dimorphic dynamics of the microtubule network during oogenesis and spermatogenesis.** (A-D′) Immunofluorescence analysis of 0 dph XX (*n*=5) (A,C,C′) and XY (*n*=3) (B,D,D′) gonads for acetylated α-tubulin and γ-tubulin (A,B), or pan α-tubulin, γ-tubulin and VASA (C-D′). Images in A and B show a ventral view of the gonads (dashed lines). Insets show oogonium (A) or spermatogonium (B) harboring the MT domes (arrowheads). Arrowheads indicate the MT domes formed around γ-tubulin-positive centrosomes. (E-N′) Immunofluorescence of 10 dph XX ovaries (*n*=7) (E-I′) and adult XY testes (*n*=4) (J-N’). Dashed lines indicate single germ cells (E,E′,J,J′) or single cysts of germ cells (F-I′,K-N’). Lower panels show *z*-stacked grayscale images of acetylated α-tubulin. The MT dome was observed in type-I oogonia (E,E′) and type-A spermatogonia (J,J′). The structure was maintained in type-II oogonia and oocytes until the zygotene stage (F-H′), whereas it was destabilized in type-B spermatogonia (K,K′). In pachytene oocytes, microtubules were organized into a cage-like structure encompassing the nucleus (I,I′). The MT dome was not observed in primary spermatocytes (pachytene) (L,L′), secondary spermatocytes (M,M′) or spermatids (N,N′). Acetylated α-tubulin signals in N,N′ show forming sperm flagella. The number of spermatogonia or cysts observed in testes (J-N′) are shown in Table S1. OC, oocytes; OG, oogonia; SC, spermatocytes; SG, spermatogonia. Scale bars: 30 μm (A,B); 10 μm (C-N′).

In a previous transcriptome analysis, we showed that the expression of genes related to the microtubule regulatory pathway changes significantly downstream of *foxl3* (Kikuchi et al., 2019). This led us to hypothesize that the organization of the microtubule network in germ cells differs between the sexes during medaka gametogenesis. To explore the microtubule network in germ cells, we labeled microtubules with antibodies against pan and acetylated α-tubulins. Both antibodies detected a conspicuous dome-shaped structure localized at the perinuclear cytoplasmic region in both oogonia and spermatogonia (Fig. 1A-D′). Co-staining with γ-tubulin and construction of a 3D image revealed that the MT dome is hollow and forms around centrosome(s), which were always located at the base of the MT dome (Fig. S1; Movie 1).

We then tracked the structure of the MT dome during gametogenesis. During oogenesis in medaka, a single stem-type germ cell (type-I oogonium) differentiates to form an oogonial cyst composed of 8-32 cells (type-II oogonia) after three to five rounds of mitosis with incomplete cytokinesis (Saito et al., 2007; Nakamura et al., 2010). The MT dome was observed at consecutive stages from type-I oogonia to oocytes at the zygotene stage (Fig. 1E-H′; Movies 2-4). The MT domes in mitotically dividing type-II germ cells in a single cyst were connected by a bundle of acetylated microtubules through intercellular bridges (Fig. 1F,F′; Movie 2). This connection was maintained until the leptotene stage, but was lost by the zygotene stage (Fig. 1G-H′; Movies 3, 4). From the pachytene stage onward, acetylated microtubules were arranged in a cage-like structure that surrounded the nucleus (Fig. 1I,I′; Movies 5, 6).

During spermatogenesis in medaka, a single stem-type germ cell (type-A spermatogonium) mitotically proliferates to form a cyst containing 128-1024 differentiating spermatogonia (type-B spermatogonia) (Shibata and Hamaguchi, 1988; Sumita et al., 2022). As in females, the MT dome was detected in type-A spermatogonia (Fig. 1J,J′). However, it was destabilized in type-B spermatogonia (Fig. 1K,K′). When type-B spermatogonia entered meiosis, acetylated microtubules transiently reappeared in primary spermatocytes, but were lost in secondary spermatocytes (Fig. 1L-M′). The microtubule structure in primary spermatocytes differed from that of the MT dome in type-A spermatogonia; it was more obscure and distributed broadly in the cytoplasm. The microtubule network is required for chromosomal arrangement during meiotic prophase I, which facilitates synapsis between homologous chromosomes (Scherthan, 2001; Chikashige et al., 2006; Shibuya et al., 2014; Mytlis et al., 2022). Thus, the transient increase of microtubules in medaka primary spermatocytes may be related to the progression of meiosis. During spermiogenesis, acetylated microtubules were strongly detected on the forming flagella (Fig. 1N,N′). All the cysts at the same spermatogenic stages (Table S1) showed the same patterns. Collectively, these findings suggest that, although the MT dome is formed in stem-type germ cells of both sexes, it is maintained only in female germ cells until the zygotene stage of meiotic prophase I. These observations indicate that regulation of MT dome stabilization differs according to sex.

### The MT dome is initially formed in PGCs and maintained in gonocytes and stem-type germ cells

**Fig. 2.**
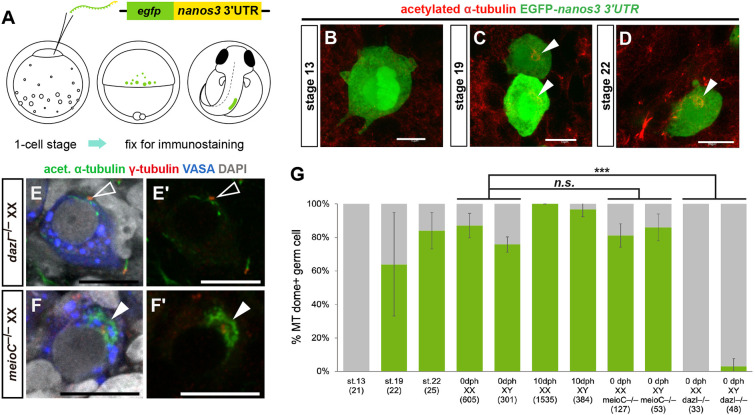
**The MT dome is formed in PGCs and is lost in gonocytes of *dazl* mutants.** (A) PGCs were labeled with EGFP by injecting *egfp*-*nanos3* 3′-UTR mRNA into one-cell-stage embryos. (B-D) Immunohistochemistry of embryos in stage 13 (B; early gastrula stage, *n*=4), stage 19 (C; two-somite stage, *n*=4) and stage 22 (D; nine-somite stage, *n*=4) for acetylated α-tubulin and EGFP. Arrowheads indicate the MT domes. (E-F′) The MT dome was not observed in *dazl*^−/−^ XX germ cells (E,E′, unfilled arrowheads; *n*=4), but was retained in *meioC*^−/−^ XX germ cells (F,F′, filled arrowheads; *n*=6) at 0 dph. (G) Quantification of the number of germ cells containing the MT dome in developing gonads of wild type and mutants. Because gonadal sex differentiation begins in stage 33, the genetic sex of stage 13-22 embryos was not specified. For 0-10 dph XX samples, only mitotic germ cells were counted. Numbers in parentheses indicate the number of germ cells quantified from *n*=4 (stage 13-22, 0-10 dph WT XY, *meioC*^−/−^ XY, *dazl*^−/−^ XX), *n*=5 (0 dph WT XX) or *n*=6 (10 dph WT XX, *meioC*^−/−^ XX, *dazl*^−/−^ XY) gonads. Data are shown as mean±s.d. ***P<0.001 (two-tailed *t*-test). n.s., not significant. Scale bars: 10 µm.

Next, we analyzed the developmental time course of MT dome formation in the germline. PGCs were labeled with EGFP by injecting synthetic *egfp* mRNA fused to the *nanos3* 3′-UTR (*egfp*-*nanos3* 3′-UTR) into one-cell-stage embryos (Fig. 2A; Kurokawa et al., 2006; Kikuchi et al., 2020). These embryos were immunostained with antibodies against EGFP and acetylated α-tubulin. At embryonic stage 13 (early gastrulation stage), when the germ cell lineage is established (Kurokawa et al., 2006), there were no PGCs with the MT dome (Fig. 2B,G). At stage 19 (two-somite stage), when PGCs are posteriorly migrating along the lateral plate mesoderm (Nakamura et al., 2006), the MT dome was observed in approximately 64% of PGCs (Fig. 2C,G). The percentage of PGCs containing the MT dome increased to 84% by stage 22 (nine-somite stage) when PGCs are much closer to the presumptive gonadal area, and the structure was consistently observed in embryonic gonocytes at the time of gonad formation (stages 30-33) and gonadal sex determination (stages 33-35) (Fig. 2D,G; Fig. S2). By 10 days post-hatching (dph), when female germ cells have entered meiosis but male germ cells continue mitotic cell division (Nishimura and Tanaka, 2014), most XX and XY mitotic germ cells, including stem-type germ cells, had the MT dome (Fig. 2G; Fig. S3). Therefore, the MT dome first appears in PGCs by stage 19 and is maintained in both XX and XY gonocytes and stem-type germ cells.

### The persistence of the MT dome requires *dazl* at the time of gonocyte development

In some vertebrates, including mammals and fish, *dazl* (*deleted in azoospermia like*) and *meioC* (*meiosis specific with coiled-coil domain*) are involved in gonocyte formation and gametogenesis commitment, respectively (Gill et al., 2011; Abby et al., 2016; Soh et al., 2017; Nishimura et al., 2018). In medaka, *dazl* mutant germ cells that reach the gonads fail to develop as gonocytes and remain in PGC-like states at the hatching stage (0 dph), whereas in the absence of *meioC* function, stem-type germ cells accumulate and no cystic or meiotic germ cells develop in the gonads (Nishimura et al., 2018). To determine whether *dazl* or *meioC* is involved in the persistence of the MT dome, we used *dazl*^−/−^ and *meioC*^−/−^ medaka at 0 dph, when germ cells that reach the gonads proliferate mitotically (Saito et al., 2007). The number of germ cells containing the MT dome significantly decreased in *dazl*^−/−^ XX and XY gonads, whereas there was no significant reduction in the number of germ cells with the MT dome in *meioC*^−/−^ compared with the wild type (Fig. 2E-G). These results suggest that *dazl* is required for the persistence of the MT dome at the time of gonocyte development in the medaka gonad, which can be discriminated from a pathway of commitment to gametogenesis by *meioC*.

### Somatic *dmrt1* is involved in destabilization of the MT dome in differentiating spermatogonia

**Fig. 3.**
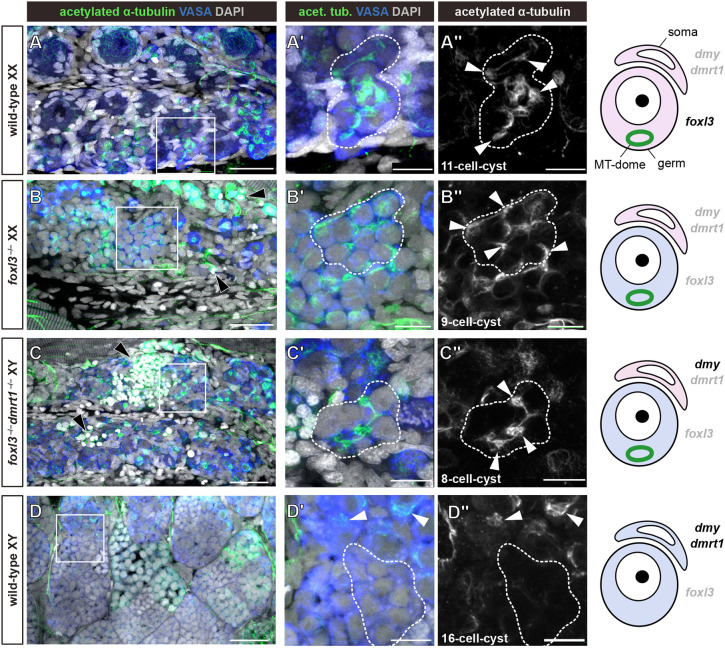
**Somatic *dmrt1* is involved in destabilization of the MT dome in differentiating spermatogonia.** (A-D″) Immunofluorescence analysis of 10 dph ovaries of wild-type XX (A-A″, *n*=5), *foxl3*^−/−^ XX (B-B″, *n*=3) and *foxl3*^−/−^, *dmrt1*^−/−^ XY (C-C″, *n*=5), and adult testes of wild-type XY (D-D″, *n*=4). Germ cells and MT domes were stained for VASA (blue) and acetylated α-tubulin (green), respectively. All confocal images are *z*-stacked to show a single cyst indicated by dashed lines. Higher magnifications of the areas boxed in A-D are shown in A′-D′ and A″-D″. The cyst size is indicated in the bottom left of A″-D″. The rightmost column illustrates the sexual fate of somatic cells (soma) and germ cells (germ). In the schematics, pink and blue indicate female- and male-fated cells, respectively. Black or gray letters indicate the genes that are ‘expressed’ or ‘not expressed’, respectively. In XX (A,B), the MT dome was maintained independently of a *foxl3* pathway. In the *foxl3*/*dmrt1* double mutant XY (C), the MT dome was observed in masculinized *foxl3*^−/−^ germ cells, whereas it was destabilized in wild-type type-B spermatogonial cysts (D). Black arrowheads in B,C indicate examples of spermatids. White arrowheads in A″-C″ indicate the MT domes in type-B germ cells. White arrowheads in D′,D″ indicate the MT domes in type-A spermatogonia. Scale bars: 30 µm (A-D); 10 µm (A′-D′,A″-D″).

In medaka, the sexual fate of germ cells is determined by *foxl3*. Loss-of-function mutants of *foxl3* produce functional sperm in XX ovaries, indicating that *foxl3* intrinsically suppresses spermatogenesis in germ cells independently of the somatic sex (Nishimura et al., 2015). To investigate whether *foxl3* is involved in MT dome stabilization in differentiating type-II oogonia, *foxl3*^−/−^ XX ovaries were immunostained with acetylated tubulin. Similar to wild-type XX, both MT domes and their microtubule connections within single cysts were observed in the *foxl3*^−/−^ XX germ cells that had committed to spermatogenesis (Fig. 3A-B″). This indicates that *foxl3* is not necessary for the formation or maintenance of the MT dome. In addition, analysis of the mutant indicated that the presence of the MT dome does not impede the progression of spermatogenesis occurring in *foxl3*^−/−^ ovaries (Fig. 3B-B″; Nishimura et al., 2015).

In wild-type testes, *dmrt1* is expressed in Sertoli cells and is required for gonadal masculinization. Depletion of *dmrt1* causes upregulation of female somatic cell markers, including *foxl2* and *aromatase*, resulting in male-to-female sex reversal of XY fish (Masuyama et al., 2012). We hypothesized that somatic *dmrt1* is involved in the negative regulation of the MT dome in XY germ cells. To explore this possibility, the MT dome was examined in *dmrt1*/*foxl3*-double knockout (dKO) XY gonads, in which *dmrt1*-depleted somatic cells become feminized, whereas *foxl3*-depleted germ cells intrinsically commit to spermatogenesis. Consistently, spermatogenesis was observed in the double mutant ovaries owing to the loss of *foxl3* in germ cells (tiny nuclei of sperm indicated by black arrowheads in Fig. 3C). We found that the MT dome remained stabilized in type-B spermatogonia of *dmrt1*/*foxl3*-dKO XY (Fig. 3C-C″). This genetic analysis indicates that *dmrt1* in somatic cells regulates a downstream pathway that intercellularly promotes destabilization of the MT dome in wild-type XY germ cells (Fig. 3D-D″).

### The MT dome enriched with organelles is a prerequisite for formation of the Balbiani body

**Fig. 4.**
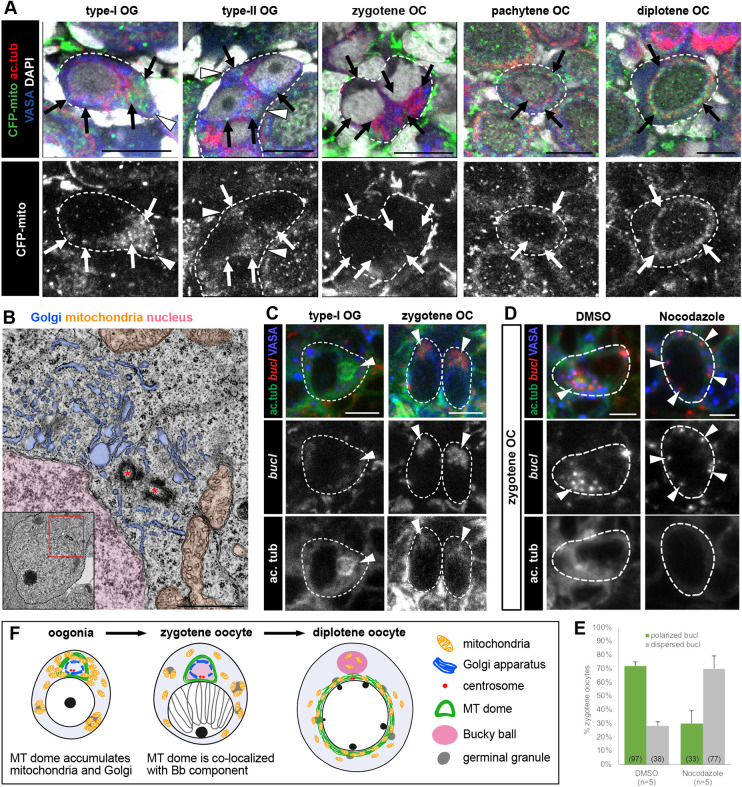
**The MT dome is located in an organelle-rich region and is enriched with *bucky ball* mRNA.** (A) Immunohistochemistry of 10 dph XX gonads (*n*=5) for CFP-mito, acetylated tubulin, and VASA. CFP-mito signal is shown in grayscale in the lower panels. Mitochondria (arrows) were accumulated around the MT domes (arrowheads) in type-I and type-II oogonia, dispersed in the cytoplasm of zygotene oocytes, and localized in the MT cage in pachytene and diplotene oocytes. Images are single-optical slices taken with a confocal microscope. Dashed lines indicate single germ cells (type-I OG, pachytene OC, diplotene OC) or single cysts (type-II OG, zygotene OC). (B) Transmission electron microscopy image of type-I oogonia at 10 dph. The cytoplasmic region indicated by a red box in the inset is magnified. Golgi apparatus, mitochondria and nucleus are colored blue, orange and pink, respectively. Centrosomes are indicated by red asterisks. (C) Co-staining of acetylated α-tubulin and *bucl* mRNA in type-I oogonia and zygotene oocytes in 10 dph XX gonads (*n*=4). Arrowheads indicate the MT domes. Dashed lines indicate single germ cells. Accumulation of *bucl* mRNA was detected from the zygotene stage. (D) Accumulation of *bucl* mRNA around the MT dome was normally detected in DMSO-treated zygotene oocytes (left; *n*=5), but was dispersed in 10 µM nocodazole-treated oocytes at the same stage (right; *n*=5). Arrowheads indicate the *bucl* signals. Dashed lines indicate single germ cells. (E) Quantification of the *bucl* mRNA localization defects shown in D. Numbers in parentheses indicate the total number of zygotene oocytes quantified from five gonads. Data are shown as mean+s.d. (F) Graphical summary of the MT dome (green), γ-tubulin (red), mitochondria (orange), Golgi apparatus (blue) and *bucky ball* (pink) localization during oogenesis. In oogonia, mitochondria and the Golgi apparatus accumulate around the MT dome as well as germinal vesicles (gray). In zygotene oocytes, the position of the Golgi apparatus is retained, but mitochondrial accumulation around the MT dome is lost. *bucky ball* mRNA colocalizes with the MT dome at the zygotene stage. By the diplotene stage, the MT dome is replaced with the Bb, and microtubules are organized into a cage-like structure around the nucleus. OC, oocytes; OG, oogonia. Scale bars: 10 µm (A); 1 μm (B); 5 µm (C).

Because the MT dome is maintained specifically in females, we speculated that the structure is associated with oocyte development. In *Drosophila* and mice, microtubule-mediated organelle transport among differentiating cyst cells is essential for oocyte development (Röper and Brown, 2004; Lei and Spradling, 2016; Niu and Spradling, 2022). We found that mitochondria labeled with a cyan fluorescent protein (CFP) tagged with a mitochondrial targeting signal (CFP-mito) were enriched around the MT dome in type-I and type-II oogonia (Fig. 4A). Transmission electron micrographs of type-I oogonia and zygotene oocytes showed that Golgi stacks were arranged in a circular pattern around centrosomes, at the location where the MT dome is supposed to form (Fig. 4B; Fig. S4). From zygotene to diplotene stages, the mitochondrial signal became dispersed in the cytoplasm and then localized to a cage-like microtubule structure around the nucleus (Fig. 4A). The timing of the loss of mitochondrial accumulation correlated with the loss of the microtubule extension from the MT domes (Fig. 1H,H′). These observations are consistent with previous reports in *Drosophila* and mice and support the hypothesis that the MT dome may act to facilitate organelle transport among female cyst cells.

In oocytes, we also found that the MT dome was a prerequisite for formation of the Bb, a conserved perinuclear aggregate of organelles and ribonucleoproteins (Billett and Adam, 1976; Heasman et al., 1984; Kloc and Etkin, 1995; Kosaka et al., 2007; Pepling et al., 2007). Bucky ball (Buc) proteins and transcripts localize to and function in Bb assembly in zebrafish, stabilizing ribonucleoproteins to the Bb (Marlow and Mullins, 2008; Bontems et al., 2009; Heim et al., 2014; Elkouby et al., 2016). In medaka, Buc is encoded by the *buc* and *bucl* genes (Figs S5, S6). Whole-gonad *in situ* hybridization showed that *bucl* mRNA was initially located in zygotene oocytes and accumulated around the Bb in previtellogenic oocytes (Fig. S7). Consistent with a report in zebrafish (Elkouby et al., 2016), *bucl* mRNA was asymmetrically localized in the cytoplasm of zygotene oocytes (Fig. 4C). In addition, the *bucl* mRNA colocalized with the MT dome in zygotene oocytes (Fig. 4C). To confirm the replacement of the MT dome with the Bb, the MT dome in oocytes was disrupted by treating 5 dph XX larvae with 10 µM nocodazole or dimethyl sulfoxide (DMSO) for 10 h. Nocodazole effectively disrupted the MT dome, showing a small remnant in zygotene oocytes, whereas the MT dome remained intact in DMSO-treated oocytes (Fig. S8). The percentage of zygotene oocytes with polarized distribution of *bucl* mRNA decreased in nocodazole-treated ovaries (30.0±9.5%; mean±s.d.) compared with that in DMSO-treated controls (71.9±3.0%) (Fig. 4D,E). These results demonstrate that the MT dome contributes to the asymmetric localization of *bucky ball* transcripts in zygotene oocytes, a key regulator of Bb formation (Fig. 4F).

## DISCUSSION

**Fig. 5.**
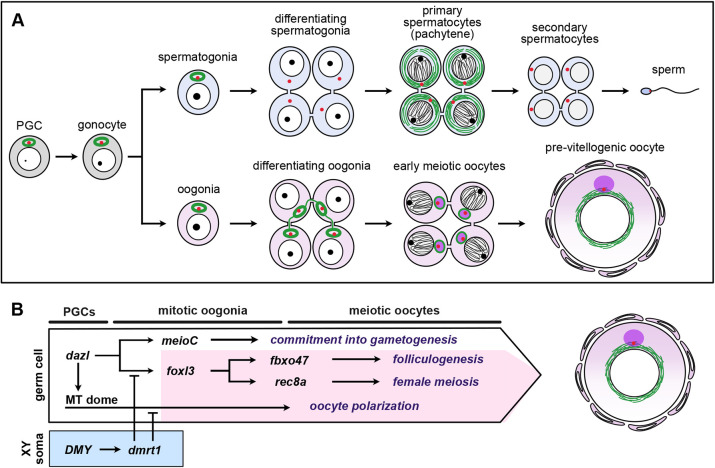
**Graphical summaries of germline sexual development and genetic pathways regulating oogenesis in *Oryzias latipes*.** (A) Sexually dimorphic changes in the microtubule network during gametogenesis. The MT dome (green oval) is formed in PGCs and maintained in gonocytes and germline stem cells. During oogenesis, the MT dome persists until the zygotene stage, and is replaced with Bb components such as *bucky ball*. By contrast, the MT dome is abolished in differentiating type-B spermatogonia. Acetylated α-tubulin (green), the centrosome (red) and the Bb (purple) are shown. (B) Genetic pathways regulating oogenesis. Each pathway contributes to a distinct feature of oogenesis, including commitment to gametogenesis, folliculogenesis, female meiosis, and oocyte polarization. In this study, we identified a pathway that regulates the MT dome, which may underlie oocyte polarity. *dazl* is required for the persistence of the MT dome in gonocytes, whereas *dmrt1* acting in XY somatic cells promotes destabilization of the MT dome in male germ cells.

This study identified a unique and conspicuous microtubule structure in medaka germ cells that is formed early during somite formation, when PGCs migrate toward gonads. Three-dimensional analysis of the structure revealed a dome-like shape, and centrosomes were detected inside the MT dome (Fig. S1). After PGCs reached the gonad, stem-type germ cells in ovaries and testes maintained the MT dome. However, the MT dome disappeared from spermatogonia at the time of differentiation, suggesting its importance in oogenesis (Fig. 5A).

The importance of the microtubule structure in oocyte development has been suggested in various animals, such as *Drosophila* (Cox and Spradling, 2003; Röper and Brown, 2004), zebrafish (Elkouby et al., 2016; Mytlis et al., 2022) and mice (Lei and Spradling, 2016; Niu and Spradling, 2022). However, genetic factors that regulate sex-based differences and dynamic changes in microtubule arrangements remain elusive in vertebrates. Contrary to our initial expectations, persistence of the MT dome during oogenesis did not require *foxl3*, which is essential for germline stem cells to commit to oogenesis (Nishimura et al., 2015; Fig. 5B). Instead, we genetically demonstrated that *dmrt1*, which acts as a masculinizer in gonadal somatic cells (Masuyama et al., 2012; Zhang and Zarkower, 2017), contributes to loss of the MT dome in germ cells during spermatogenesis (Fig. 5B). In the absence of *dazl*, germ cells that reached the gonad failed to retain the MT dome (Fig. 2E,E′), suggesting that *dazl* function is required for the persistence of the MT dome at the time of development of PGCs into gonocytes. The present data also suggest that *meioC*, which is important for germ cells to commit to gametogenesis (Abby et al., 2016; Nishimura et al., 2018), is not required for the presence of MT dome in type-I germ cells (Fig. 2F,F′), although the possibility remains that *meioC* is involved in maintaining the MT dome in differentiating oogonia. Taken together with a previous report that *meioC* and *foxl3* are expressed downstream of *dazl* (Nishimura et al., 2018), the findings suggest that the MT dome pathway toward Bb formation functions in parallel with the *meioC* and *foxl3* pathways (Fig. 5B).

On the basis of previous reports and the present data, there are three possible explanations for the role of the MT dome. First, we showed that the MT dome in medaka is positioned in an organelle-enriched area and extends microtubule bundles toward adjacent germ cells in a cyst via intercellular bridges (Figs 1F-G′ and 4A). This association disappears as intercellular connections are lost during meiotic prophase I. This implies that the MT dome in medaka functions as a center for microtubule extension connecting different cystic germ cells and is consequently involved in organelle and cytoplasmic transfer. This is consistent with previous studies in *Drosophila* and mice showing microtubule-dependent organelle transport among ovarian cystic germ cells via intercellular bridges (Röper and Brown, 2004; Lei and Spradling, 2016; Niu and Spradling, 2022).

A previous study found that the fertilization rate of *foxl3*^−/−^ sperm is lower than that of the wild type (Nishimura et al., 2015). This may be due to the persistence of the MT structure for differentiating spermatogonia in *foxl3*^−/−^ovaries, although the presence of the MT dome did not impede the progression of spermatogenesis (Fig. 3B-B″). The presence of the MT dome may affect the quality of sperm.

Second, our observation that disruption of the MT dome by nocodazole results in dispersion of *bucl* mRNA in zygotene oocytes suggests that the MT dome is important for recruiting this Bb regulator and possibly for Bb assembly. Disruption of the MT dome also resulted in localization of the nucleus to the center of the cells, in contrast to the wild type in which the nucleus was asymmetrically positioned in cytoplasm (Fig. 4D). Thus, the MT dome may be a prerequisite for the acquisition of oocyte polarity by predetermining the location, and it accordingly would set the foundation for the Bb. In zebrafish, the Bb localizes mRNAs such as dorsal determinants and related factors vegetally in oocytes (Nojima et al., 2010; Lu et al., 2011; Ge et al., 2014), and the lack of Bb results in symmetrical eggs that fail to develop beyond the early cleavage stages (Marlow and Mullins, 2008). It would be interesting to track the development of nocodazole-treated oocytes to investigate the role of the MT dome in animal–vegetal axis formation in medaka.

Third, the MT dome may be involved in meiotic chromosome movement. In zebrafish, zygotene oocytes and spermatocytes form a cilium that is involved in telomere clustering via stabilization of the centrosome position (Mytlis et al., 2022). Although we were unable to detect the cilial structure in medaka zygotene meiocytes of both sexes, it is possible that the MT dome in zygotene oocytes (Fig. 1H,H′) plays a similar role in stabilizing centromeres, thereby clustering the telomeres. In males, the MT dome became undetectable in mitotically active type B spermatogonia (Fig. 1K,K′), although acetylated microtubules reappeared at the pachytene stage (Fig. 1L,L′), suggesting its potential involvement in chromosome movement during meiotic prophase I in medaka.

In summary, we showed that a germ cell-specific microtubule structure is first recognized in PGCs, dynamically changes its structure in a sex-specific manner, and finally leads to the development of the Bb, an important factor in oocyte polarization (Fig. 5A). Genetic analyses showed that the male-specific disruption of the MT dome in germ cells was mediated by a pathway involving somatic *dmrt1*, but not germ cell *foxl3*. This adds a new female-specific pathway to the two previously identified pathways triggered by *foxl3* (Fig. 5B). The present results suggest that germ cells are intrinsically directed to become oocytes, as suggested by the acquisition of oocyte-specific polarity from their primordial state.

## MATERIALS AND METHODS

### Fish

The OKcab strain of medaka fish (*Oryzias latipes*) was used in this study. Fish were maintained in fresh water at 25-28°C under photoperiodically regulated conditions (14 h light and 10 h dark). Developmental stages of embryos were determined according to Iwamatsu (2004). All experiments were conducted with the approval of the Nagoya University official ethics committee (Approval Number S220008-002 in Department of Science, Nagoya University). Genotypic sex of all animals was determined by qPCR using TaqMan MGB probes (Thermo Fisher Scientific) that detect the male-determining gene *DMY* (Matsuda et al., 2002; Nanda et al., 2002) and the autosomal gene *cyp19a1* as described previously (Kikuchi et al., 2020). Generation and identification of the mutant alleles of *dazl* (ex2-Δ34) (Nishimura et al., 2018), *meioC* (ex5-Δ25) (Nishimura et al., 2018) and *foxl3* (NΔ17) (Nishimura et al., 2015) were described previously.

### Generation of *dmrt* (Δ13) mutant by TALEN-induced mutagenesis

The transcription activator-like effector nuclease (TALEN) target sites for *dmrt1* exon 2 were searched using the TALEN Targeter program (https://tale-nt.cac.cornell.edu/node/add/talen) with the following parameters: spacer length of 15-18 bp, repeat array of 16-18 bp, and upstream base of T only. TALEN assembly was performed following a modified version of the original protocol (Sakuma et al., 2013). *dmrt1*-target sites were as follows: right, TTCCGGAGGGCCCGGC; left, GTCCCCCCGGATGCCCA. TALEN plasmids were linearized by NotI digestion and used as templates for *in vitro* RNA synthesis with the mMESSAGE mMACHINE T7 transcription kit (Thermo Fisher Scientific). TALEN mRNAs (250 ng/μl left and right) were injected into one- or two-cell-stage embryos. The F0 founders were crossed with wild-type medaka. A mutant allele with 13 bp deletion (deleted sequence: GGCTCCGGCTCCA; *dmrt1*Δ13) was identified in F1 adult fish using the primer sets shown in Table S2 and sequence analysis. *dmrt1*Δ13^+/−^ fish were crossed with *foxl3*Δ17^+/−^ fish to obtain *foxl3*Δ17^+/−^; *dmrt1*Δ13^+/−^ double heterozygous fish, which were intercrossed to obtain the double-homozygous mutants used in this study.

### Immunohistochemistry (IHC)

Adult testes were isolated and fixed with 4% paraformaldehyde (PFA) [pre-warmed to room temperature (RT)] for 2 h at RT. Embryos and larvae were fixed with 4% pre-warmed PFA for 1 h at RT. Before fixation, eggs were dechorionated with hatching enzyme purchased from NBRP Medaka (https://shigen.nig.ac.jp/medaka/strain/hatchingEnzyme.jsp), and larval skin at the ventral side was opened by forceps. Fixed samples were washed five times in PTW (PBS with 0.1% Tween 20) for 5 min each wash at RT. For larval samples, the skin around gonadal region was removed by forceps and the head was cut for genomic lysate preparation for genotyping. For immunostaining, the samples were blocked in 20% Blocking One (Nacalai Tesque) in PTW for 2 h at RT, and treated with primary antibodies overnight at 4°C. After washing three times in PTW for 30 min each wash at 4°C, the samples were treated with secondary antibodies and DAPI overnight at 4°C. Immunostained samples were washed with PTW for 10 min at RT and mounted on slides with Vectashield (Vector Laboratories). Fluorescent images were acquired using an Olympus FV1000 confocal scanning microscope. Volocity imaging software was used for 3D reconstruction of confocal images.

Primary antibodies for the following proteins were used: medaka VASA (Aoki et al., 2008) (1:100; rat antiserum, lab-made), α-tubulin (1:100; mouse, Sigma-Aldrich, T6557), acetylated α-tubulin (1:100; mouse, Sigma-Aldrich, T6793), γ-tubulin (1:100; rabbit, GeneTex, GTX113286), EGFP and CFP (1:100; rabbit, GeneTex, GTX113617). Secondary antibodies used were: Alexa Fluor 488-, 568- and 647-conjugated goat antibodies (1:200; Thermo Fisher Scientific, A-11077, A-11031; Cell Signaling Technology, 4408S, 4410S, 4412S, 4414S, 4416S).

### *In situ* hybridization (ISH)

cDNAs of *buc* and *bucl* were amplified from ovarian cDNA library by PCR using T7-promoter-tagged primers (Table S2). After gel-purification, the PCR products were used as templates for *in vitro* transcription of digoxygenin (DIG)-labeled RNA probe using T7 RNA polymerase (Sigma-Aldrich) and DIG RNA Labeling Mix (Sigma-Aldrich) according to the manufacturer's instructions.

Adult ovaries were fixed with 4% PFA and dehydrated in 100% methanol as described in the ‘Immunohistochemistry (IHC)’ section. The samples were sequentially washed in 75%, 50% and 25% methanol in PTW, and twice in PTW at RT for 5 min in each. Then, the samples were treated with 10 µg/ml proteinase K (Roche) in PTW for 15 min at RT, then fixed with 4% PFA for 20 min at RT. After washing five times with PTW for 5 min each wash, the samples were pre-hybridized in hybridization buffer [50% formamide containing 5× SSC, 0.1% Tween 20, 5 mg/ml torula RNA (Sigma-Aldrich) and 100 µg/ml heparin sulfate] for 2 h at 65°C, followed by hybridization with heat-denatured DIG-labeled RNA probe diluted in hybridization buffer at 65°C overnight. Hybridized samples were washed twice with 2× SSC containing 0.1% Tween 20 (SSCT) in 50% formamide for 30 min, twice in 2× SSCT for 15 min, and twice in 0.2× SSCT for 30 min each wash at 65°C. The samples were then blocked with 5% sheep serum in PTW for 2 h at RT. The buffer was replaced by anti-Digoxigenin-AP Fab fragments (1:20,000; Merck, 11093274910) in 5% sheep serum/PTW overnight at 4°C. After washing six times with PTW for 10 min each wash and twice in staining buffer [100 mM Tris-HCl (pH 9.5), 100 mM NaCl, 50 mM MgCl_2_, 0.1% Tween 20] for 5 min, signal was detected by 2% NBT/BCIP (Merck, 11681451001) in staining buffer. Signals were observed using a KEYENCE BZ-X microscope.

### Fluorescent ISH (FISH)-IHC

For FISH-IHC, the ISH protocol was followed up to the blocking step. The samples were treated with antibodies for acetylated α-tubulin (1:100; mouse, Sigma-Aldrich, T6793), medaka VASA (Aoki et al., 2008) (1:100; rat antiserum, lab-made), and Digoxigenin-POD (1:100; Boehringer, 1207733), in 5% sheep serum at 4°C overnight. After washing three times with PTW, anti-DIG-POD signal was amplified using the TSA Biotin System Kit (Perkin Elmer) according to the manufacturer's instruction. Then, the samples were treated with secondary antibodies (1:100; anti-mouse IgG-Alexa 647, Cell Signaling Technology, 4410S; anti-rabbit IgG-Alexa 488, Cell Signaling Technology, 4412S; streptavidin-Alexa 568, Thermo Fisher Scientific, S11226) and DAPI in 5% sheep serum at 4°C overnight. Fluorescent signals were detected using an Olympus FV1000 confocal scanning microscope.

### Quantifying germ cell number and statistical analysis

For quantification of germ cells with the MT dome, EGFP-*nanos3* 3′UTR-positive or VASA-positive cells in embryos or gonads were counted, respectively. For 10 dph XX gonads, meiotic oocytes with condensed chromosomes and small nucleoli were excluded from quantification. Four to six embryos or gonads per different developmental stages were counted for quantification. Data are presented as mean±s.d. The *P*-value for the data between two groups was determined using an unpaired, two-tailed *t*-test. Statistical significance was set at *P*<0.01.

### Transmission electron microscopy

For transmission electron microscopy analysis, 10 dph XX larvae were dissected and fixed with 0.1 M cacodylic acid buffer containing 2% PFA, 2% glutaraldehyde and 0.1% picric acid. Sample preparation and image acquisition were performed by Tokai Electron Microscopy, Inc.

### Nocodazole treatment

Twenty medaka larvae at 5 dph were transferred into four wells of a 24-well plate (five larvae/well), each of which contained 1 ml reverse osmosis water supplemented with 10 µM or 100 µM nocodazole (Merck, M1404) or DMSO. Larvae were then incubated at room temperature for 2, 4, 10 or 14 h, followed by fixation in 4% PFA for IHC and FISH-IHC assays. Zygotene oocytes were identified by DAPI-stained condensed chromosomes forming a bouquet structure and by localization of nucleoli near the nuclear membrane.

## Supplementary Material



10.1242/develop.201840_sup1 Supplementary information
