# Supplementary material for: Sexually dimorphic dynamics of the microtubule network in medaka (Oryzias latipes) germ cells
Source: Development. 2024 Mar 13;151(5):dev201840. doi: 10.1242/dev.201840 (PMC10984276; doi:10.1242/dev.201840)
Supplement: Supplementary information [file develop-151-201840-s1.pdf]

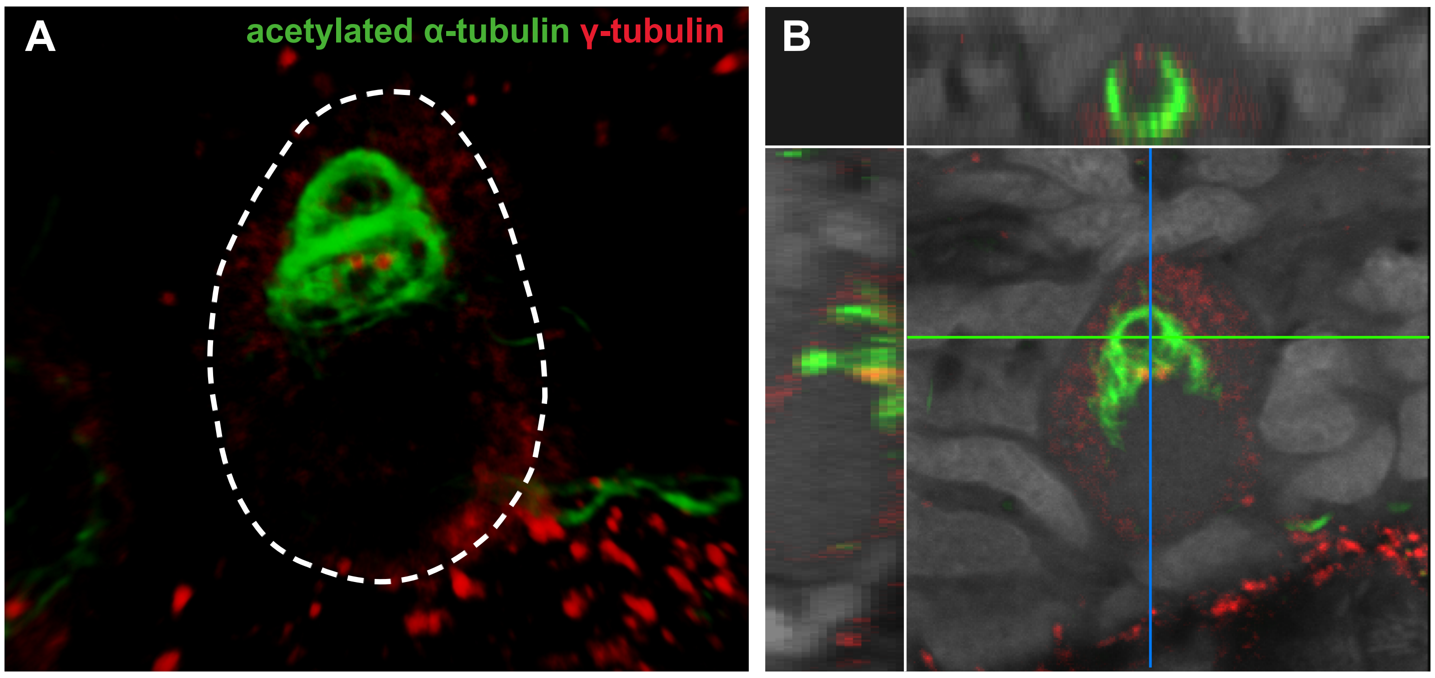

Fig. S1. (A) Three-dimensional reconstruction of a 10 dph XYgerm cell (dotted line) stained with antibodies against acetylated α-tubulin (green) and γ-tubulin (red). Centrosomes are located at the base of the MT dome. **(B)** Horizontaland vertical sections of the 3D image are shown in *A*.The MTdome has a hollow inside.

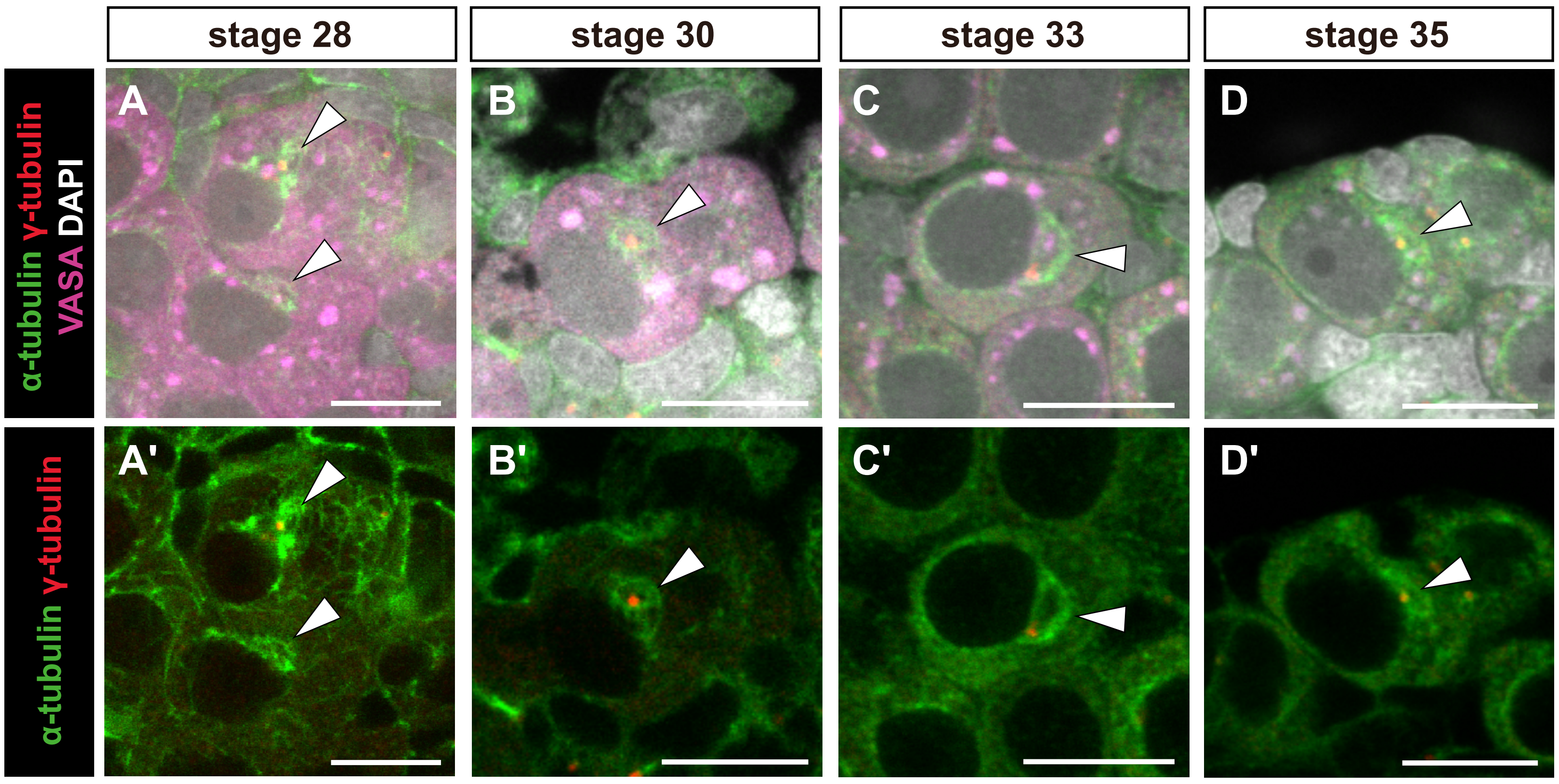

Fig. S2. Immunohistochemistry of embryos in stage 28 (*A*; 30-somite stage), stage 30 (*B*; 35-somite stage), stage 33 (*C*; notochord vacuolization stage), and stage 35 (*D*; visceral blood vessels formation stage) stained for pan α-tubulin, γ-tubulin, and VASA. In stage 30, PGCs come in touch with gonadal somatic precursors and move dorsally to the prospective gonadal region by stage 33. Expression of the sex-determining gene, *DMY/dmrt1bY*, in stage 33 triggers sexual differentiation of gonads by stage 35. Arrowheads indicate the MT domes in PGCs (*A, B*) or gonocytes (*C, D*). Scale bars: 10 µm.

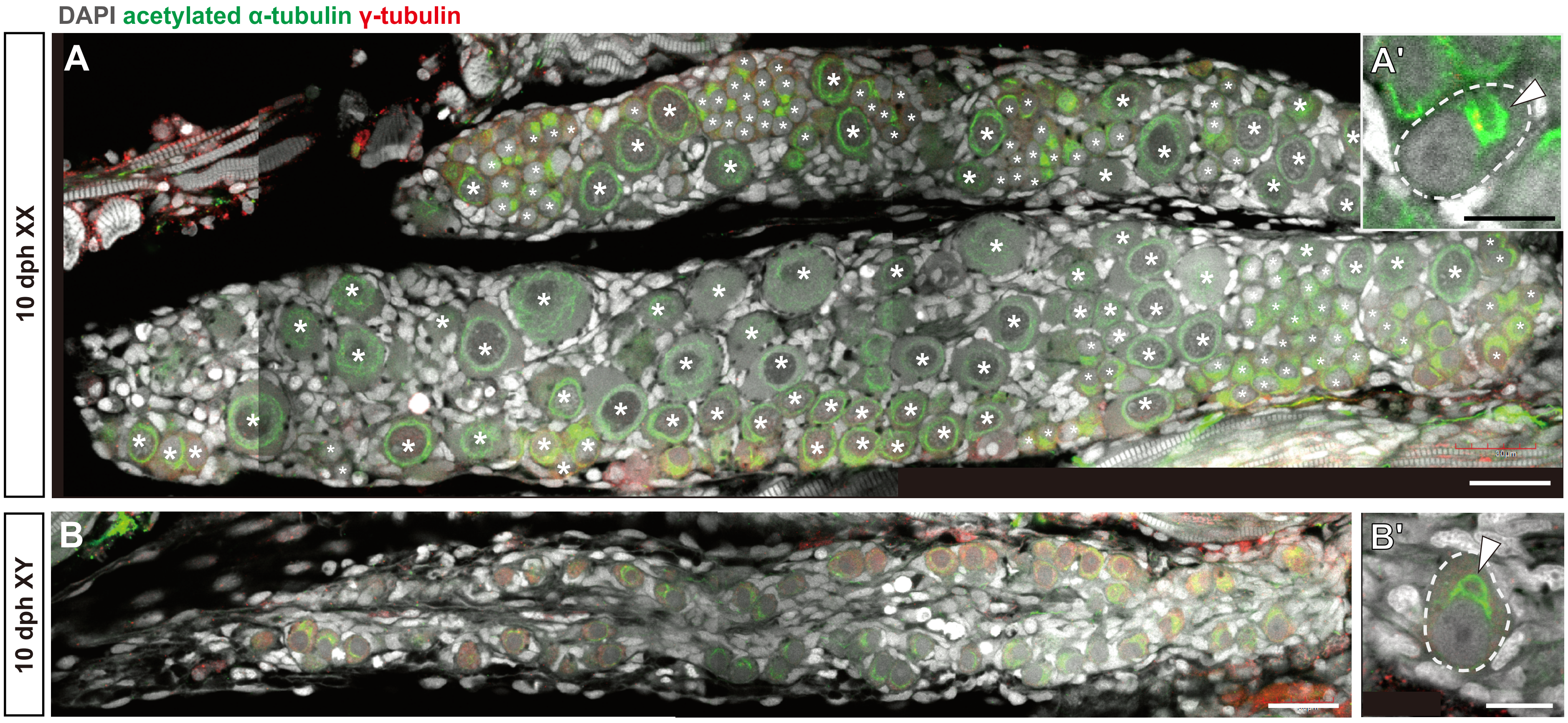

Fig. S3. Ventral views of 10 dph XX *(A)* and XY *(B)* gonads.Inthisstage,XXovariescontain not only mitotic germ cells but also many meiotic oocytes (asterisks in *A*), whereas XY testes contain only mitotic germ cells (Saito *et al.*, 2007). Magnified images of mitotic germ cells are shown in *A′* and *B′*. All mitotic germ cells possess the MT dome (arrowheads). Scale bars: 30 µm (*A, B*) or 10µm (*A′, B′*).

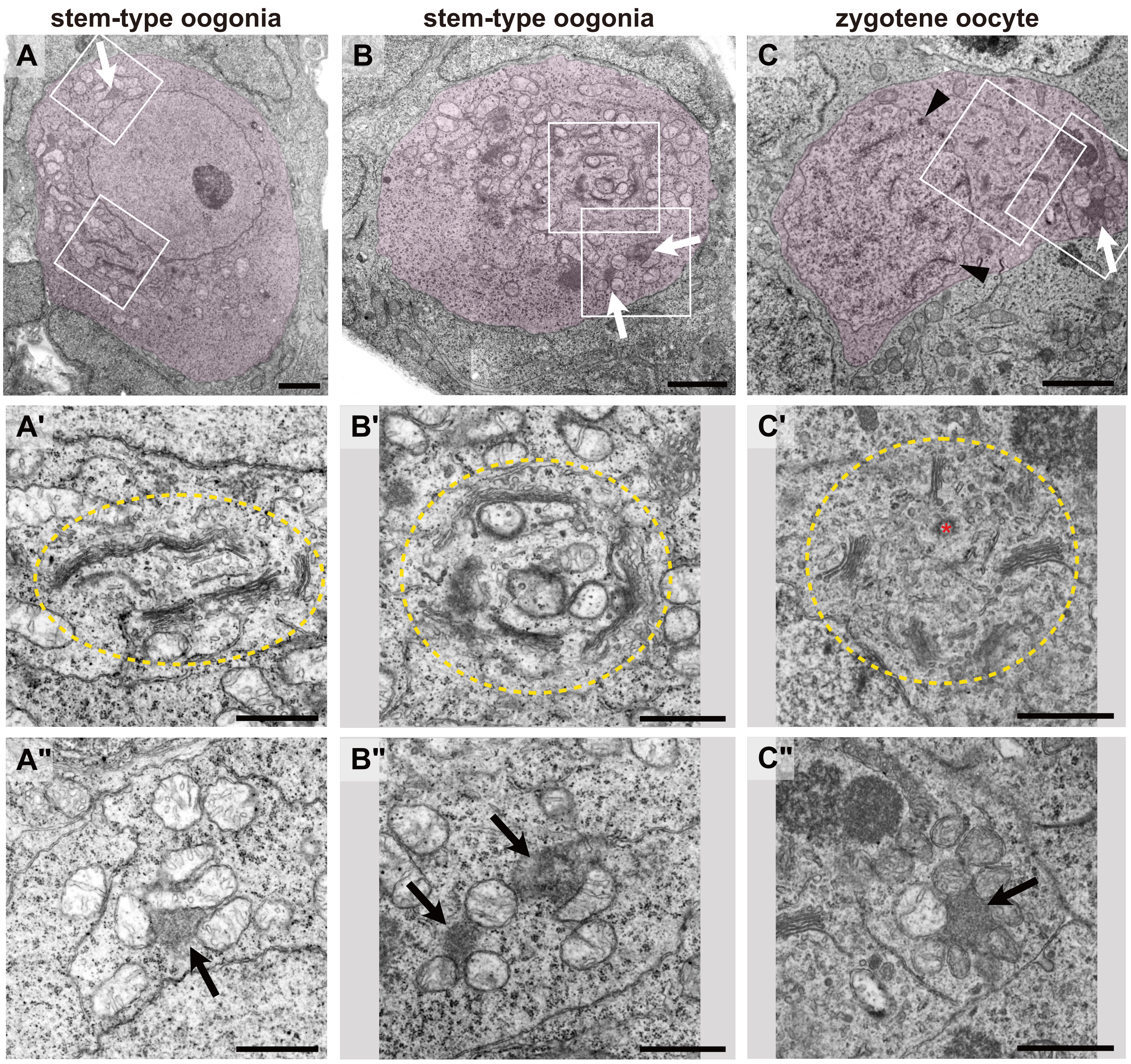

Fig. S4. TEM images of type-I oogonia (*A, B*) and a zygotene oocyte (*C*). Germ cells are colored pink. White arrows indicate germinal vesicles. Brack arrowheads in *C* indicate synapsed homologous chromosomes, a typical feature of zygotene oocytes. The areas indicated by white squares are magnified in *A′–C′* and *A″–C″*. Yellow dotted lines in *A′–C′* indicates the Golgi stacks arranged in a circular pattern. Black arrows in *A″–C″* indicate germinal vesicles. The asterisk in *C′* indicates a centrosome. Scale bars: 2 µm (*A–C*) or 1 µm (*A′–C′, A″–C″*).

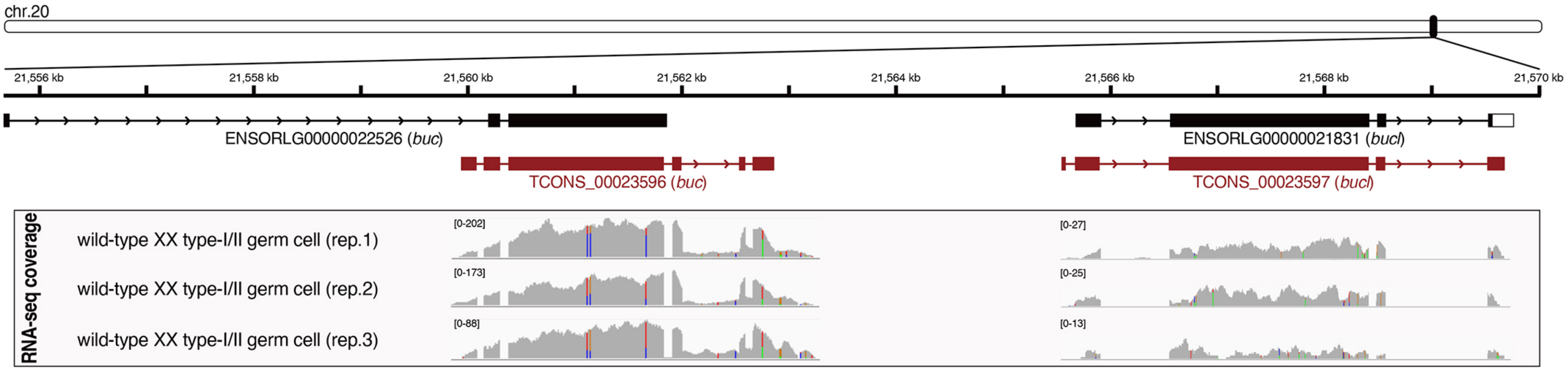

Fig. S5. Genomic loci of *bucky ball* (*buc*) and its paralog *bucky ball–like* (*bucl*). Predicted gene organizations based on Ensembl genome browser release109 (black; Cunningham et al. 2022 *Nucleic Acids Res.* 50, D988-995) and on our RNA-seq read mapping (red; Kikuchi *et al.*, 2019) are indicated. RNA-seq read coverages of medaka XX type-I/II germ cells (three replicates) for *buc* and *bucl* are shown at the bottom.

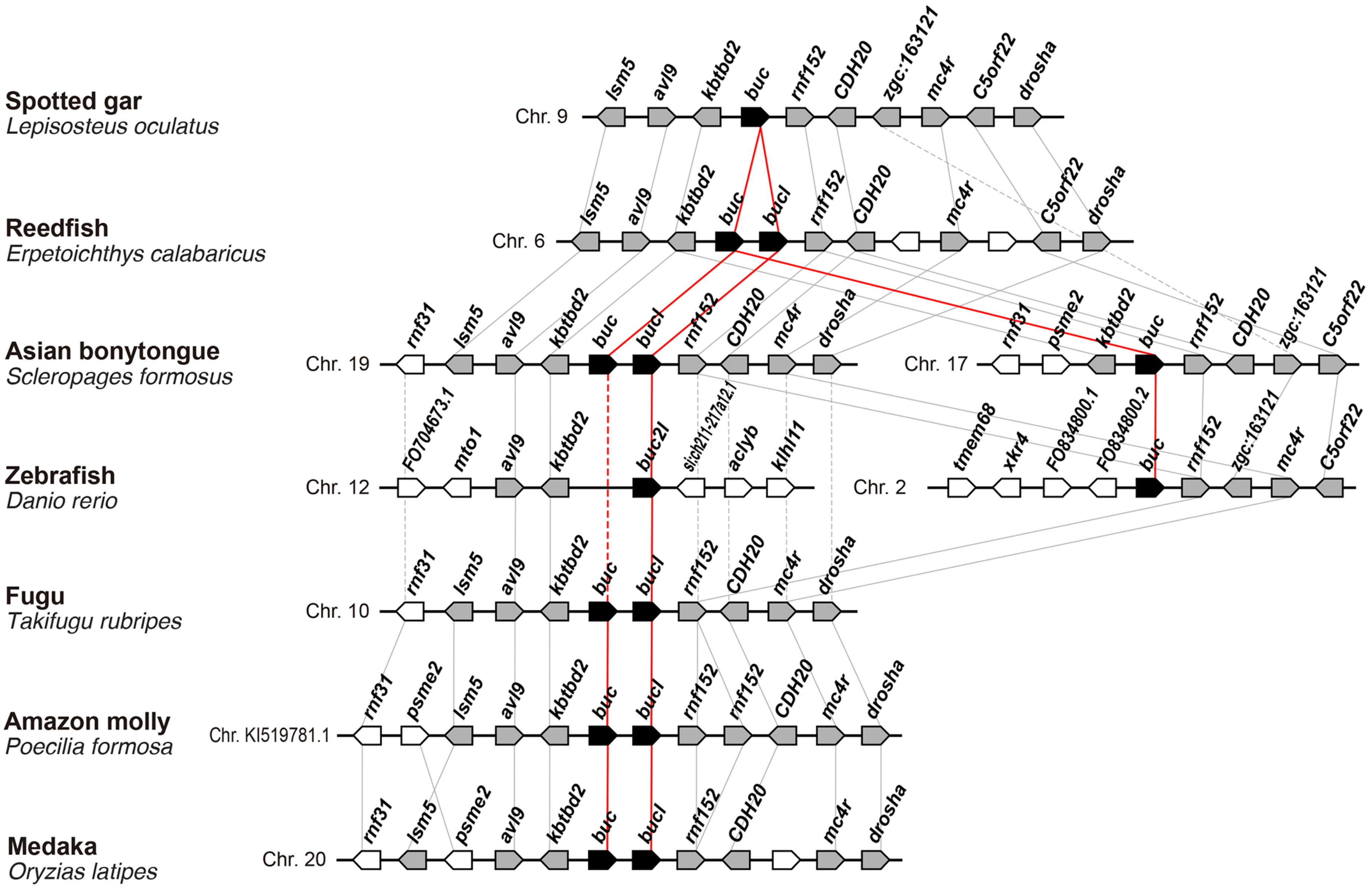

Fig. S6. Syntenic map of *buc* and its paralogs *bucl* and *buc2l* (black, blunt end: 5′). Species and chromosome are indicated on the left. Gray and red lines connect orthologs between species. The synteny of gray genes is conserved from Spotted gar.

Development • Supplementary information

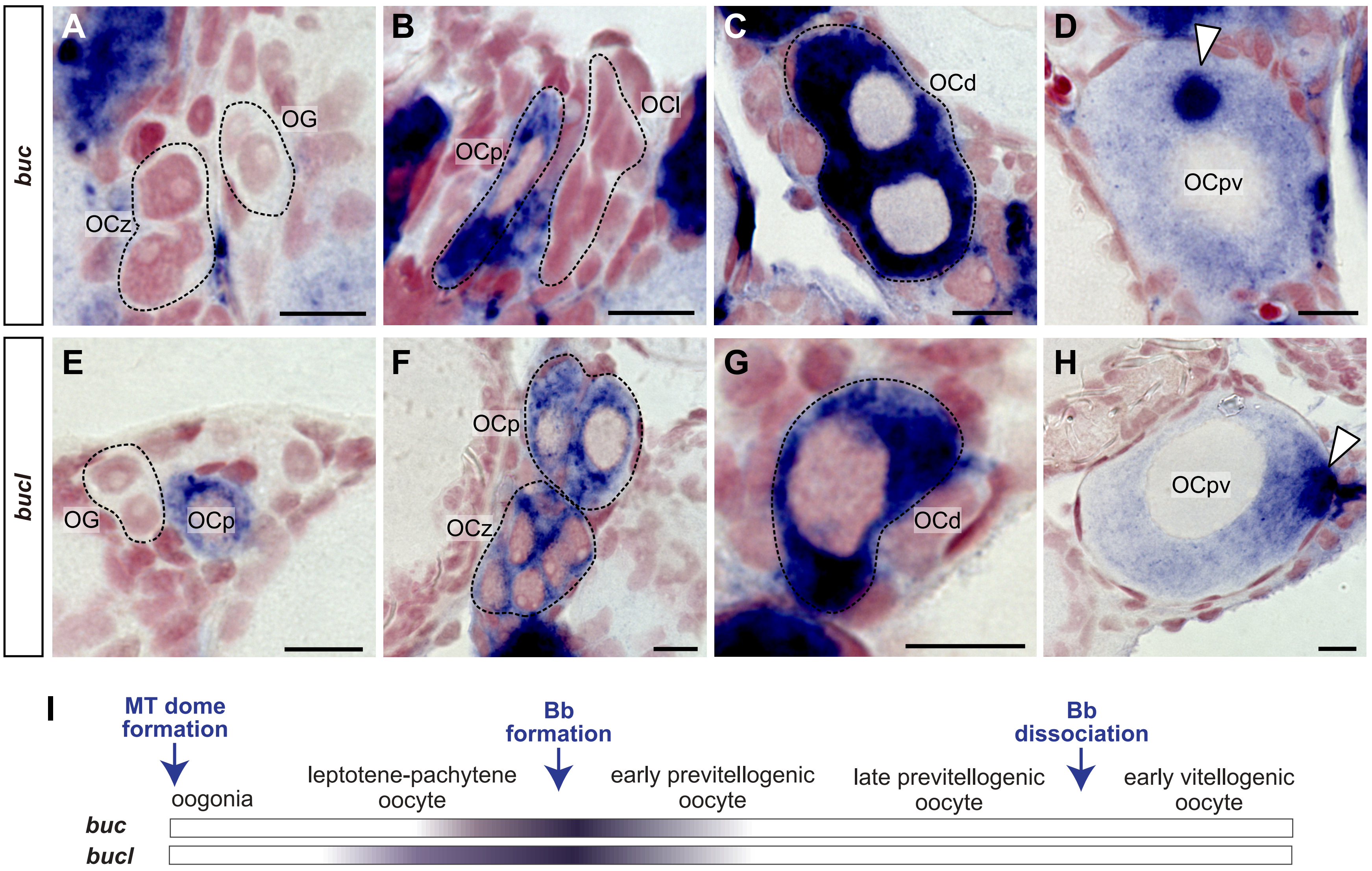

Fig. S7. *In situ* hybridization of the Bb components, *buc* (*A–D*) a n d *bucl* (*E–H*). NBT/BCIP signals are shown in blue. Expression patterns are summarized in *I*. Expression of both genes was detected in meiotic oocytes. Transcripts of *buc* and *butl* were localized to the Bb (arrowheads) in previtellogenic oocytes. OG: oogonia, OCl: leptotene oocyte, OCz: zygotene oocyte, OCp: pachytene oocyte, OCd: diplotene oocyte, OCpv: previtellogenic oocyte. Developmental stages of germ cells were determined as follows. OG: the nucleolus (faintly stained with neutral red) is located at the center of the nucleus. OCl: the nucleolus is located at the nuclear periphery. OCz: the nucleolus is located at the nuclear periphery, and condensed chromosomes are visible in the nucleus. OCp: condensed chromosomes are still visible in nucleus, and cellular size increases. OCd: chromosomes decondense, and cellular and nuclear sizes increase to up to 90 μm in diameter. OCpv: a cellular size increases up to 150 μm in diameter. Scale bars: 10 μm.

Development • Supplementary information

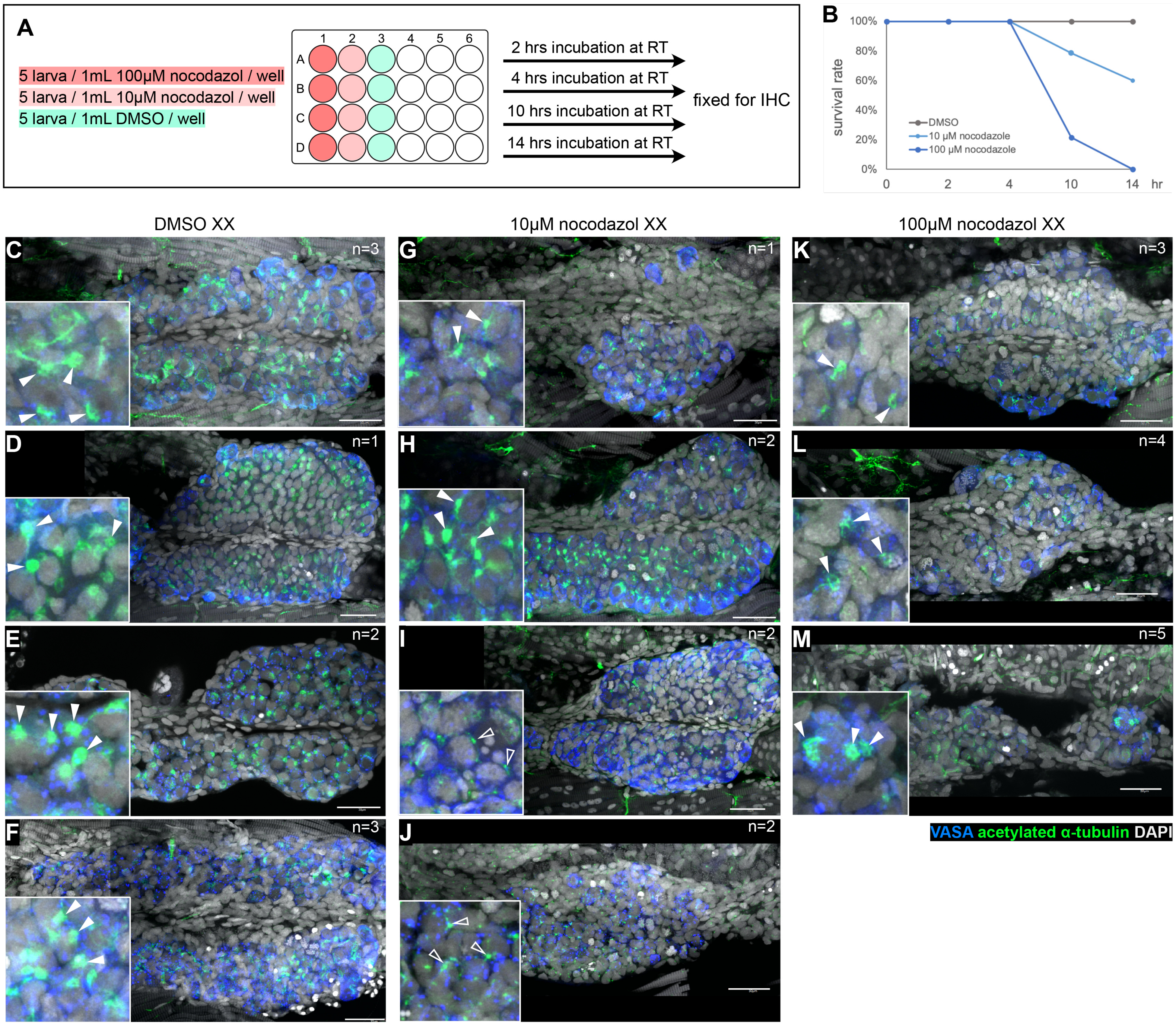

Fig. S8. Disruption of the MT dome in XX germ cells by nocodazole treatment. (*A*) Experimentalschemefor nocodazole treatment. Five larvae at 5 dph were incubated in 1 mL RO water containing 100 μM nocodazole, 10 μM nocodazole, or DMSO for 2, 4, 10, or 14 h at room temperature, and then fixed for immunohistochemistry. (*B*) Survivalrateoflarvaeduring nocodazole- or DMSO-treatment. (*C–M*) Immunohistochemistry of 5dph XX larvae treated with DMSO (*C–F*), 10 μM nocodazole (*G–J*), or 100 μM nocodazole (*K–M*) for 2 h (*C, G, K*), 4 h (*D, H, L*), 10 h (*E, I, M*), or 14 h (*F, J*). Images show the ventral view of ovaries stained for VASA (blue) and acetylated α-tubulin (green). Magnified images of zygotene oocytes are shown at the lower left. Filled and open arrowheads indicate normal and disrupted MT domes, respectively. In gonads treated with 10 μM nocodazole for more than 10 h, the MT domes were severely disrupted. The numbers of samples observed are shown in the upper right. Scale bars: 30 μm.

Table S1. The number of single germ cells or cysts observed in Figure 1J–N.

sample	# type-A SG	# type-B SG cyst	# pachytene SC cyst	# second. SC cyst	# ST cyst
1	10	10	10	10	0
2	10	10	10	6	3
3	10	10	9	9	9
4	10	10	6	6	6
total	40	40	35	31	18

Table S2. Primers used in this study

PRIMER NAME	PRIMER SEQUENCE (5' – 3')	PURPOSE
dmrt1-TALEN-Δ13-F	GGGCCCGGCTCCGGG	dmrt1Δ13 mutant genotyping
dmrt1-TALEN-R1	TTCAGCGGAGACACGAAGCC	dmrt1Δ13 mutant genotyping
dmrt1-TALEN-F2	TCCTGTACAAGTGACCCCGC	dmrt1Δ13 mutant genotyping, sequencing
dmrt1-TALEN-R2	GCAATCAGCTTGCATTTGGC	dmrt1Δ13 mutant genotyping, sequencing
buc-ISH-F	gagaaacggcctgttcagag	DIG-probe for in situ hybridization
buc-ISH-T7R	ggccagtgaattgtaatacgactcactatagggttcccacaacaagcacacat	DIG-probe for in situ hybridization
bucl-ISH-F	acctggtcacagacctcacc	DIG-probe for in situ hybridization
bucl-ISH-T7R	ggccagtgaattgtaatacgactcactatagggggaacgaagcagttctcgtc	DIG-probe for in situ hybridization

Development • Supplementary information

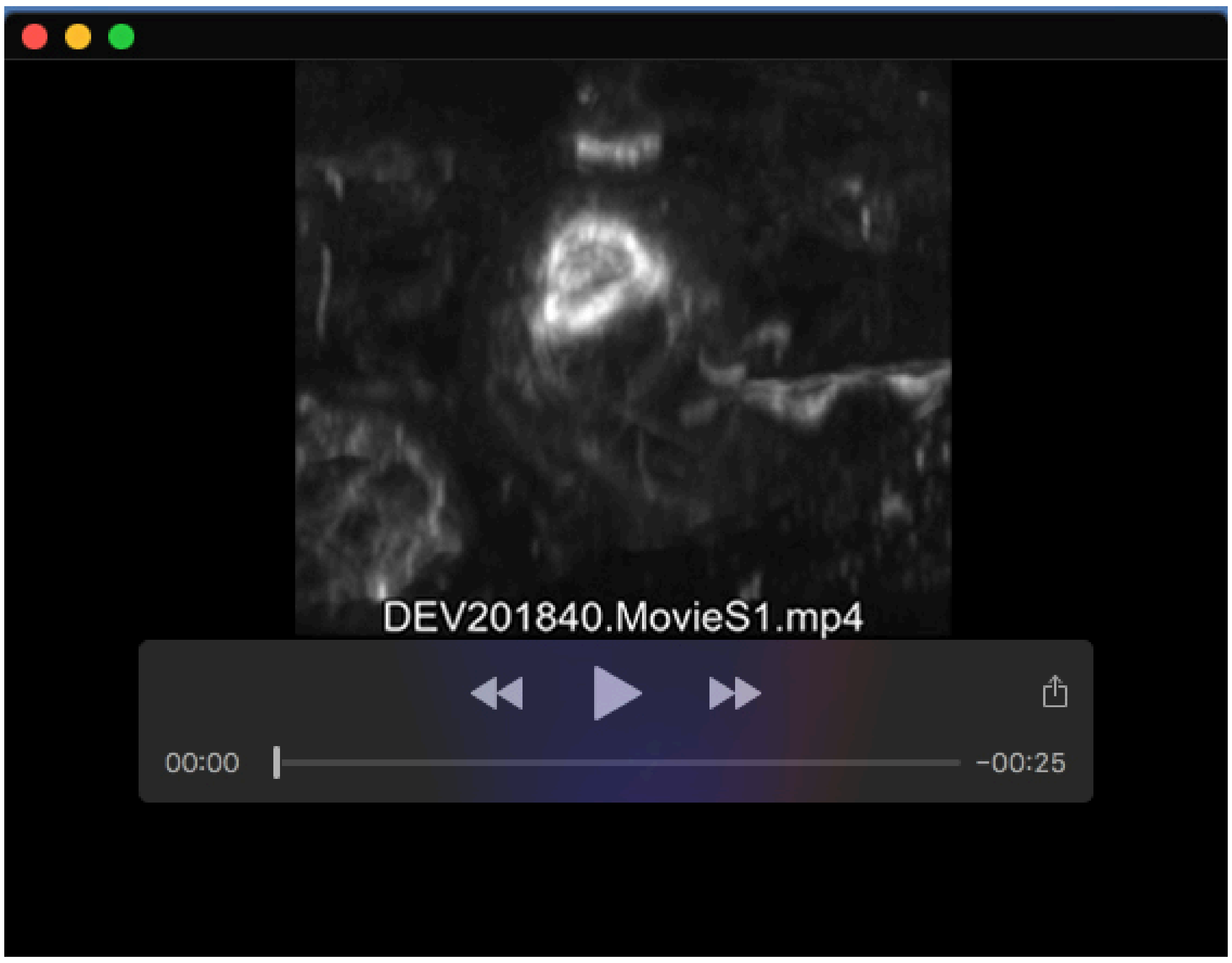

Movie 1. Three-dimensional reconstruction of acetylated α-tubulin signals in stem-type spermatogonia. The MT dome is constructed in the perinuclear cytoplasmic region. The same cell is shown in Figure S4B'.

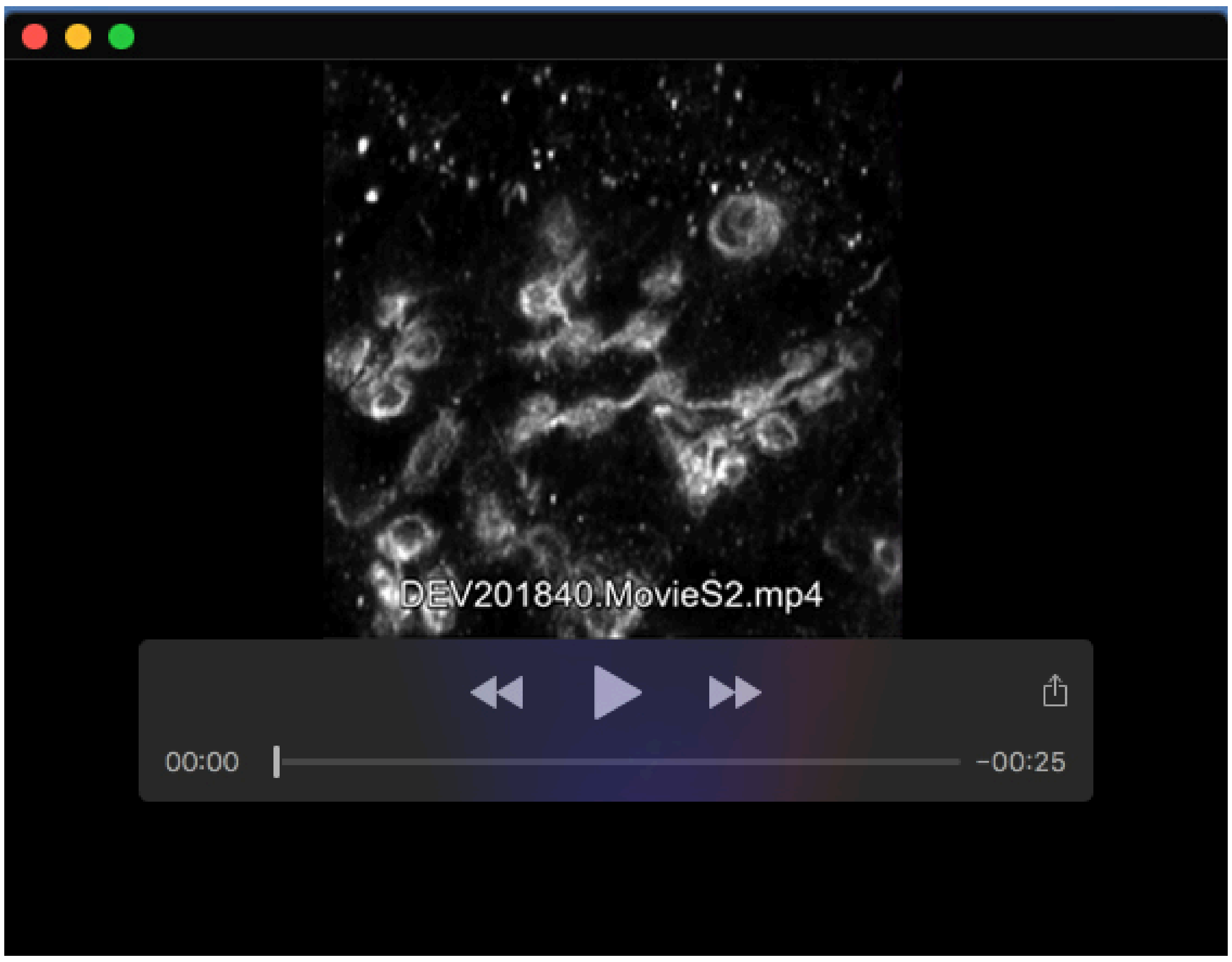

Movie 2. Three-dimensional reconstruction of acetylated α-tubulin signals in a cyst of differentiating oogonia. The MT domes are connected to each other through intercellular bridges. The same cyst is shown in Figure 1B.

Development • Supplementary information

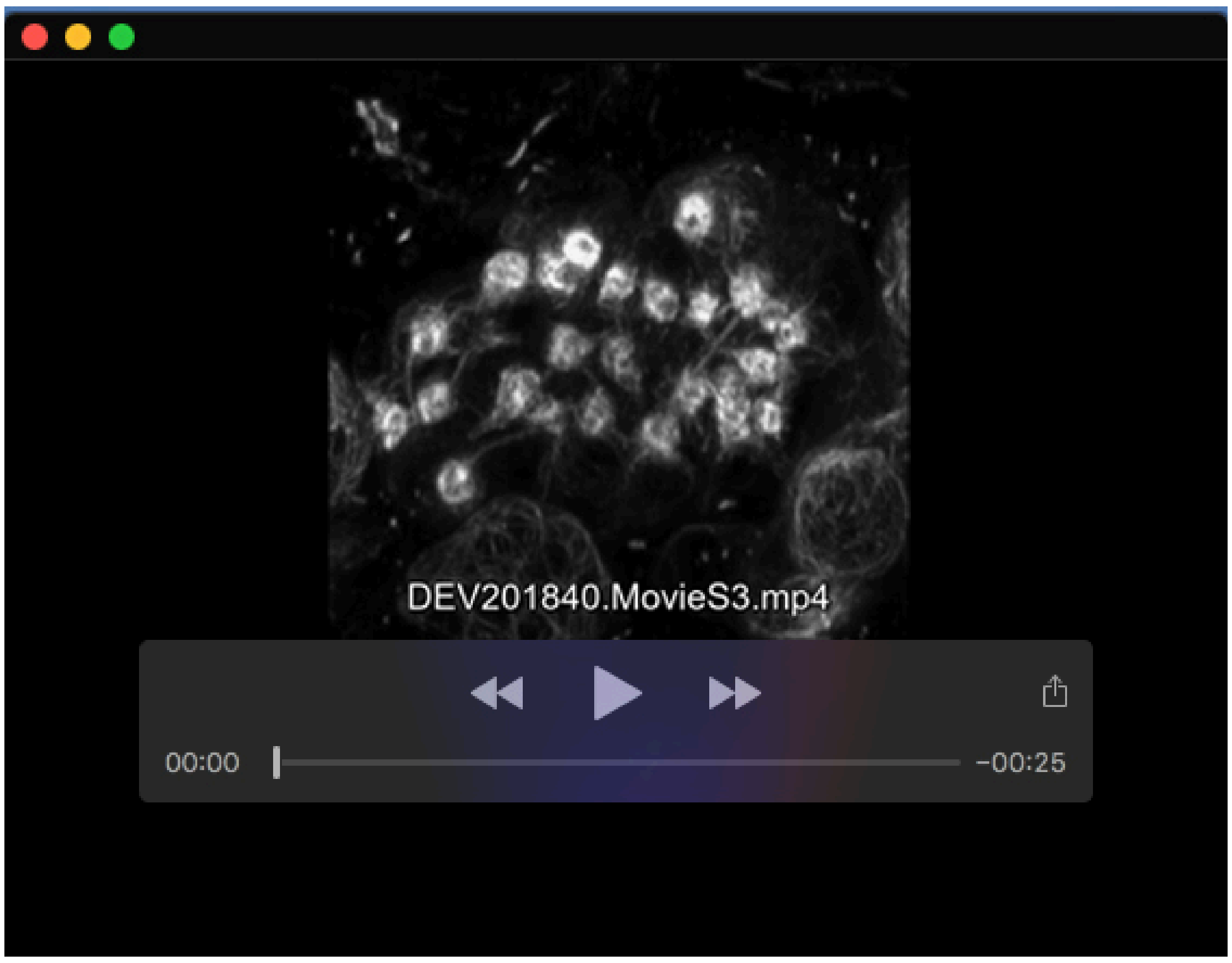

Movie 3. Three-dimensional reconstruction of acetylated α-tubulin signals in a cyst of leptotene oocytes. Microtubules connecting the MT domes were thinner than those in differentiating oogonia. The same cyst is shown in Figure 1C.

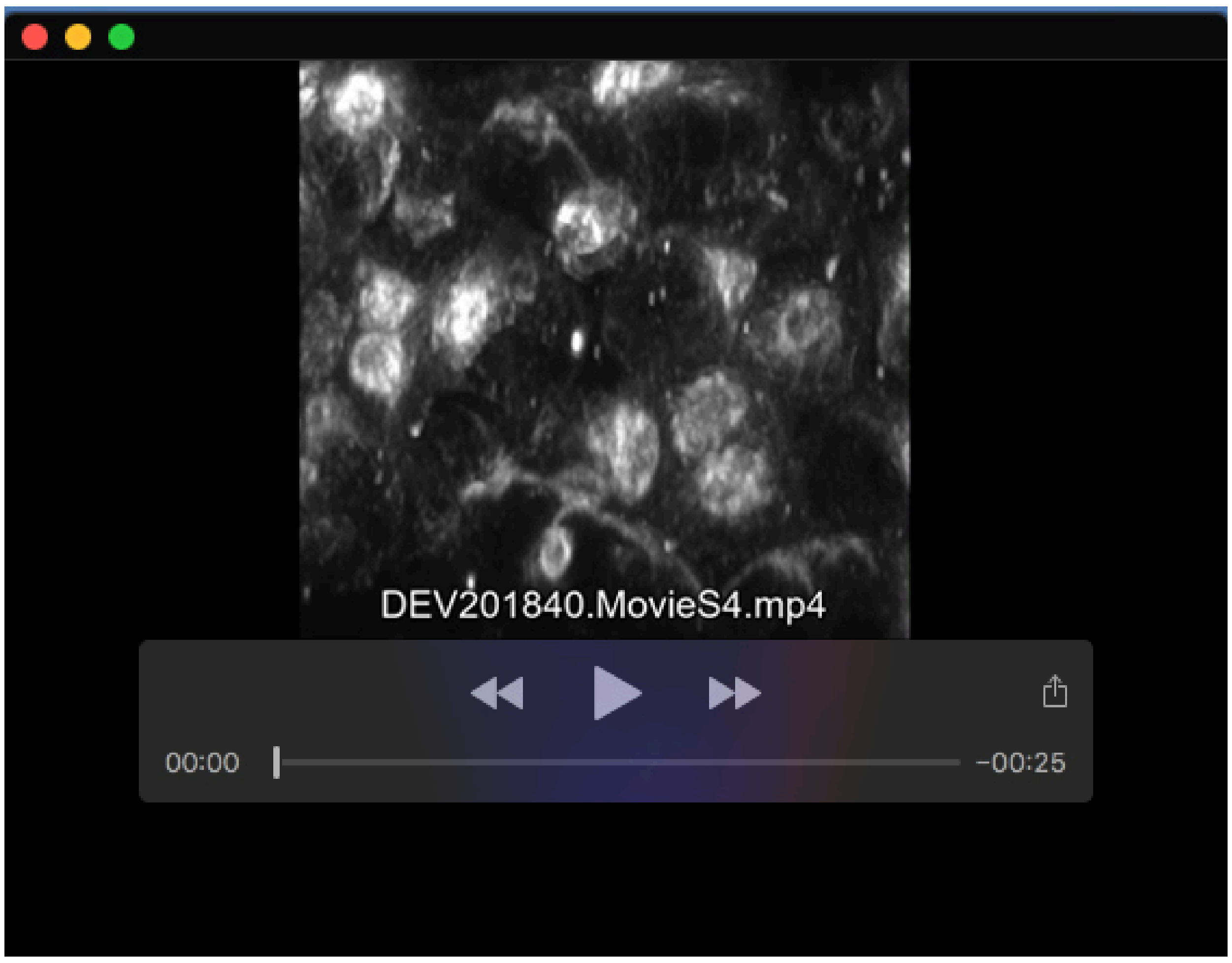

Movie 4. Three-dimensional reconstruction of acetylated α-tubulin signals in a cyst of zygotene oocytes. The MT domes were no longer connected at this stage. The same cyst is shown in Figure 1D.

Development • Supplementary information

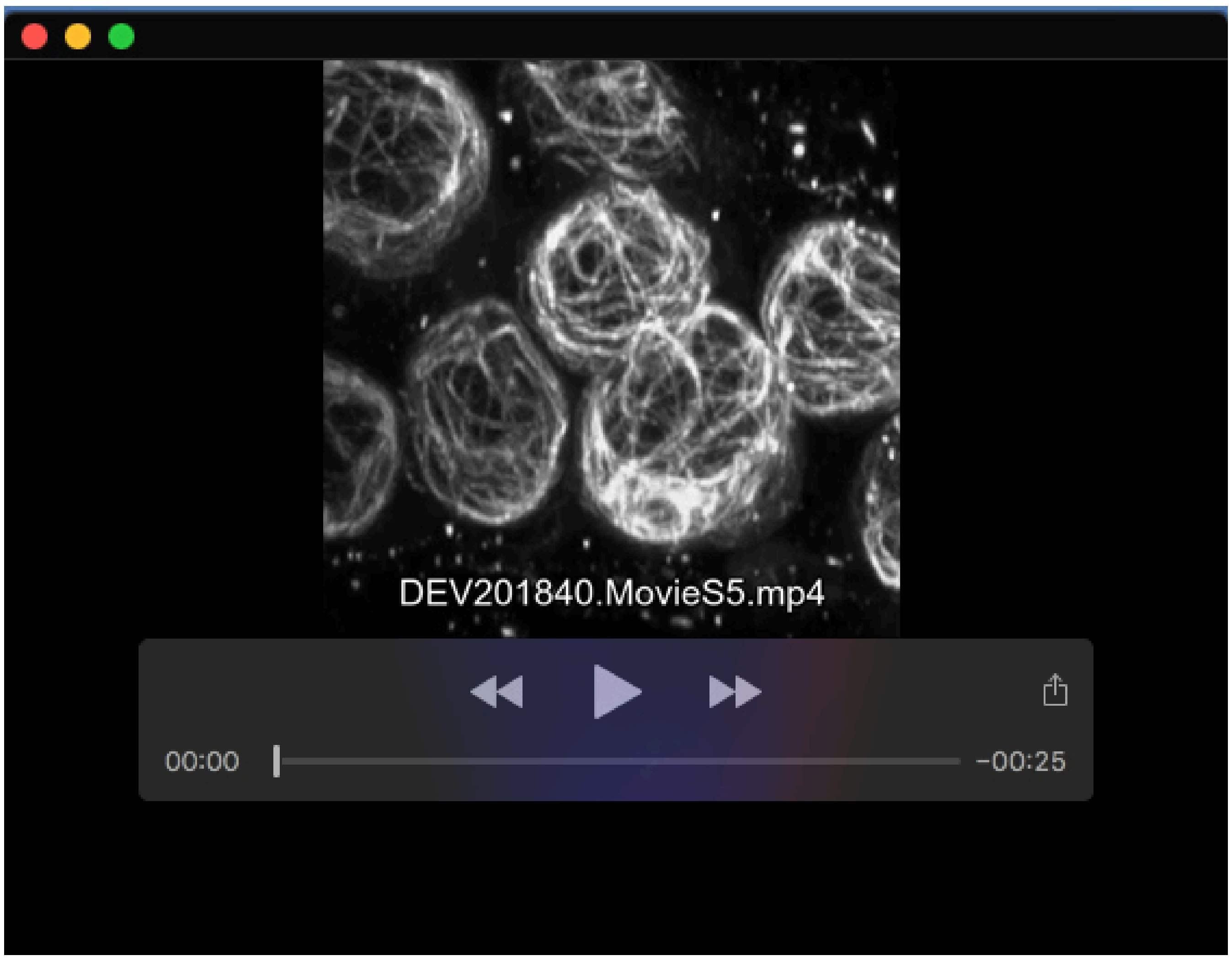

Movie 5. Three-dimensional reconstruction of acetylated α-tubulin signals in pachytene oocytes. A cage-like microtubule structure was formed around the nucleus. The same cells are shown in Figure 1E.

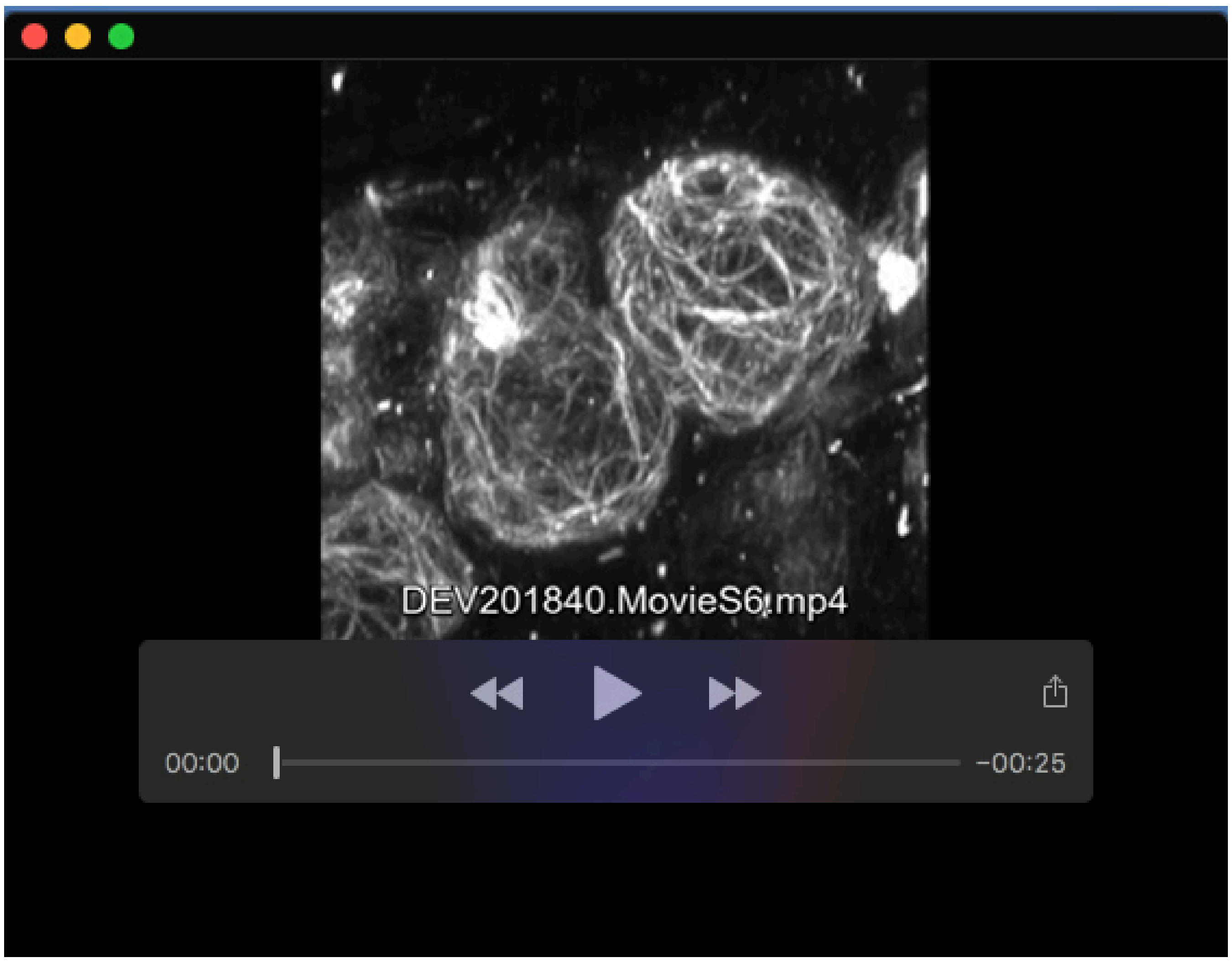

Movie 6. Three-dimensional reconstruction of acetylated α-tubulin signals in diplotene oocytes. A cage-like structure is maintained at this stage.

Development • Supplementary information
