# Supplementary material for: Sexually dimorphic dynamics of the microtubule network in medaka (Oryzias latipes) germ cells
Source: Development. 2024 Mar 13;151(5):dev201840. doi: 10.1242/dev.201840 (PMC10984276; doi:10.1242/dev.201840)
Supplement: Supplementary file 1 [file develop-151-201840_review_history.pdf]

Sexually dimorphic dynamics of the microtubule network in medaka (*Oryzias latipes*) germ cells

Mariko Kikuchi, Miyo Yoshimoto, Tokiro Ishikawa, Yuto Kanda, Kazutoshi Mori, Toshiya Nishimura and Minoru Tanaka
DOI: 10.1242/dev.201840

Editor: Swathi Arur

Review timeline

First submission:	11 April 2023
Editorial decision:	17 April 2023
Rebuttal:	4 May 2023
Rebuttal response:	12 May 2023
First revision received:	14 August 2023
Editorial decision:	8 September 2023
Second revision received:	6 January 2024
Editorial decision:	25 January 2024
Third revision received:	30 January 2024
Accepted:	5 Febraury 2024

First Submission

Decision Letter

MS ID#: DEVELOP/2023/201840

MS TITLE: Sexually dimorphic dynamics of the microtubule network in medaka (*Oryzias latipes*) germ cells

AUTHORS: Mariko Kikuchi, Miyo Yoshimoto, Toshiya Nishimura, Tokiro Ishikawa, Yuto Kanda, Kazutoshi Mori, and Minoru Tanaka

Many thanks for submitting your manuscript to Development. I have read it carefully and I am sorry to say that I am returning it without review. The identification of a polarizing center in the primordial germ cells which forms into the balbiani body in oocytes is consistent with known work from the field. In the absence of further mechanistic insights into this process, I am afraid that the guidelines to our reviewers would almost certainly lead to the rejection of the paper after the review process. I hope that this rapid decision will give you the opportunity to submit your work to a more suitable journal without further delay.

Rebuttal Letter

I appreciate your effort to spend your time reading our manuscript very carefully. I understand you have a deep insight in reproduction by studying oogenesis of nematoda.

But let me describe our opinions on your decision letter. In your letter, there are two points that have you make a decision.

First, "identification of a polarizing center in the primordial germ cells which forms into the balbiani body in oocytes is consistent with known work from the field".

© 2024. Published by The Company of Biologists under the terms of the Creative Commons Attribution License (https://creativecommons.org/licenses/by/4.0/).

We understand that many people 'feel' that a polarizing center may locate at the future place of Balbiani body. But the main point of our manuscript is not identification. As we mention below, the points are the sexual dimorphism of the microtubule structure which is first formed in PGCs in both sexes and the presence of a novel germ cell feminizing pathway which is initiated in PGCs (Figure5). But as Dr. Arur pointed out, we also 'suggest' by several supporting evidences that this structure seems replaced with Balbiani body. But we would like to emphasize that, in vertebrates, there is no direct experimental evidence showing that MTOC is replaced with Balbiani body so far we understand. Our manuscript provides a novel understanding of how germ cells with sexually identical microtubule structure at the beginning of PGC stage develop into germ cells with female-specific structure (consequently Balbiani body), which is completely a new point of view in gametogenesis (sex differentiation of germ cells).

Here let us mention the polarity in germ cells of another vertebrates briefly. The polarity is first observed in mice by visualization of microtubules at E15.5 (equivalent to zygotene stage). The paper (Niu, 2022, Science, Fig.5G) says just the presence of “synchronous cyst mitoses up to E14.5” but does not say anything about asymmetrically localized microtubules in germ cells before meiosis. In zebrafish, an uneven distribution of microtubule is first recognized in leptotene stage, followed by localization of dazl transcripts and Golgi apparatus at zygotene stage (Elukouby, 2016, PLoS Biol). But no report of the polarity before entry into meiosis and in PGCs.

Regarding the microtubule structure in our manuscript, let us raise the following two points:
1. The unknown structure that we call it MT dome is continuum of the structure which is first created at a PGC stage and is kept maintained until oocytes. Nobodies have addressed such a structure in PGCs. This continuum is a new finding never reported in vertebrates.
2. We know that there are extensive analyses providing an insight into mechanism of Balbiani formation. But there has been no report addressing why Balbiani body forms only in female germ cells. Our main focus is sexual dimorphic dynamics of the microtubule structure between the two sexes, which provides a new insight into what makes Balbiani body only in female.

Second, “In the absence of further mechanistic insights into this process”.

As mentioned above, we say with confidence that our manuscript identifies the genes leading to the dimorphic dynamics. The disruption of an oogenic pathway by somatic-expressing dmrt1 and the maintenance of the microtubule structure by dazl create dimorphic gametes (figure 5). And this identification clearly uncovers the presence of a new genetic pathway which makes germ cells oocytes and begins to run in PGCs, in addition to the previously identified two pathways triggered by foxl3. So now we can clearly say by our manuscript that there are three distinct pathways that makes germ cells oocytes. As we believe that Dr. Arur knows very well, there have been few studies in vertebrates, addressing clearly what pathways are initiated at the timing of commitment to oogenesis or spermatogenesis. Identifying another pathway in vertebrates is a great progress. Our results by genetic analyses clearly shows the new mechanistic insight and the presence of pathway leading to oogenesis.

I fully understand ‘Development’ demands and prefer to fine mechanisms but I also know that there are many articles accepted that reveal new pathways 'by genetics' if they demonstrates a clear insight into the mechanisms.

Since we believe our results contribute a lot to new understanding of gametogenesis, we chose 'Development'. So, we are very happy to have any explanation acceptable to us for our opinion. We appreciate your precious time you will spend and sincerely hope that our manuscript will be reconsidered for a review process.

Rebuttal Decision Letter

Thank you for your appeal on your recently rejected manuscript. I do understand your disappointment.However, we are always willing to give authors the chance to defend their manuscripts, and I do recognise that you make some valid comments in your letter. Therefore we

© 2024. Published by The Company of Biologists under the terms of the Creative Commons Attribution License (https://creativecommons.org/licenses/by/4.0/).

would be willing to reconsider a revised version of your manuscript that deals, as far as possible, with the points that were raised by me in the initial rejection letter. Specifically, as far as possible address the points in the manuscript that you submit for review and spell out in the abstract as well as the body of the paper, the novelty of the genetic mechanisms that you have identified.

I will solicit reviewer comments on the manuscript, once I receive it.

First revision

Author response to reviewers' comments

In response to our rebuttal letter, the editor allowed us to resubmit our paper, considering our main claims in our original paper. The editor initially thought that a main contention of our original manuscript is analysis of Balbiani formation in oocytes. But we explained that it was not our intention but focuses on a novel structure of microtubule and sexually different regulation of the structure. Since there were not specific comments in the editor's reply, we describe below our revised points in the resubmitted paper. The same is also stated in Cover Letter.

Our manuscript has been largely and carefully revised according to the points below.
1. Sexually different changes of microtubule structure (MT dome) during gametogenesis. The MT dome first appears in PGCs in both sexes.
2. Presence of a novel pathway implementing oocyte-unique character
3. Accordingly, 'Summary Statement' is modified so that 'sexual difference' of the pathways is emphasized.
As mentioned in our previous letter, identification of a polarizing center (Balbiani body: Bb) is not our main claim. How the polarizing center is developed specifically in oogenesis but not in spermatogenesis is one of our main contentions. Discussion on Bb, however, still occupies some portion in our manuscript because we think audience will wonder what the MT dome means.

More details about revision:
In 'Introduction', we described characters unique to oocytes, advantage to use medaka, Bb, and a brief summary of our findings. As mentioned above, Bb is described so as to address a point of our manuscript: how it is formed only in oogenesis.
'Discussion' begins with a brief summary of MT dome development, then moves to our discovery of a novel pathway leading to sexual difference of this structure. Next, the roles of the MT dome are described with previous reports by other researchers and discussion is focused about our identification of genetic factors that promotes novel understanding of sexual difference of the microtubule structure during gametogenesis. Lastly, we describe several pathways (<I>foxl3</I>-dependent and independent) that implement oocyte-unique characters, which initiate at different time points of gametogenesis.

Second decision letter

MS ID#: DEVELOP/2023/201840

MS TITLE: Sexually dimorphic dynamics of the microtubule network in medaka (*Oryzias latipes*) germ cells

AUTHORS: Mariko Kikuchi, Miyo Yoshimoto, Toshiya Nishimura, Tokiro Ishikawa, Yuto Kanda, Kazutoshi Mori, and Minoru Tanaka

I have now received all the referees' reports on the above manuscript, and have reached a decision. The referees' comments are appended below, or you can access them online: please go to BenchPress and click on the 'Manuscripts with Decisions' queue in the Author Area.

© 2024. Published by The Company of Biologists under the terms of the Creative Commons Attribution License (https://creativecommons.org/licenses/by/4.0/).

As you will see, the referees express considerable interest in your work, but also provide recommendations to significantly improve the rigor of the claims made in the study. Specifically as suggested by reviewer 1 and 3, clear experimental evidence would be needed to support the role of the microtubule dome in males but not female germ cells, and its role in sexual differentiation. If you are able to revise the manuscript along the lines suggested, which involve further experiments, I will be happy receive a revised version of the manuscript. Your revised paper will be re-reviewed by one or more of the original referees, and acceptance of your manuscript will depend on your addressing satisfactorily the reviewers' major concerns. Please also note that Development will normally permit only one round of major revision.

Please attend to all of the reviewers' comments and ensure that you clearly highlight all changes made in the revised manuscript. Please avoid using 'Tracked changes' in Word files as these are lost in PDF conversion. I should be grateful if you would also provide a point-by-point response detailing how you have dealt with the points raised by the reviewers in the 'Response to Reviewers' box. If you do not agree with any of their criticisms or suggestions please explain clearly why this is so.

Reviewer 1

Advance summary and potential significance to field

In many animals, including mammals, gonad development begins with sexually indifferent early germ cells and somatic gonad precursors that form a sexually indifferent gonad that later differentiates as ovary or testis. The mechanisms that regulate specification of the early germline and later sexual differentiation of the gonad vary widely but involve factors, many of which are conserved, that trigger molecular and cellular events that regulate gonad differentiation as ovary or testis. Understanding the earliest cellular and molecular events in sexual differentiation of the gonads is an interesting and significant problem. In this manuscript from Kikuchi and colleagues, the authors examined microtubule organization in developing medaka germ cells from embryonic stages through early gonad formation and differentiation as ovary or testis and identified a sexual dimorphism in the pattern of microtubule organization in mitotic germ cells.
Specifically, they observed microtubules asymmetrically localized in mitotic cysts of sexually undifferentiated gonads that form dome structures and connect cells within cysts. They observed that these microtubule domes and connections persist in ovaries, but not in differentiated testis. They make use of previously established mutant lines to explore the relationship between these microtubule domes and connections and early germline development (dazl mutants) and sex determination (foxl3 and dmrt1 mutants). This analyses confirms the ovary and testis specific patterns and presence of microtubule domes. In terms of ovary development, the beautiful images show asymmetric microtubule structures at stages of development before asymmetry has been reported thus far. Using gamma tubulin as a marker of centrosomes, they show that microtubule domes form around these microtubule organizing centers at stages before earlier oocyte asymmetries, the Balbiani body specifically, form. To examine the relationship between microtubule domes and Balbiani body formation, they examine acetylated microtubules and the localization of transcripts encoding Medaka homologs of a regulator of Balbiani body development in zebrafish, bucky ball. Because domes form first and buc later localizes proximal to the centrosome, the authors conclude that microtubule domes are precursors to the Balbiani bodies and later polarity that forms in oocytes.

Overall, this is an exciting observation, and the authors have attempted to determine the molecular hierarchy regulating sexual dimorphic maintenance of domes and potential roles of the domes in oocytes. However, the paper as written does not place the exciting findings in context and is difficult to understand in part due to a need for an introduction that better sets up the question(s) and places the work in the context of the field(s) – sex determination on one hand and oocyte patterning on the other. There are also potentially confusing or misleading sentences and grammatical errors that need to be addressed, as well as incomplete or inconsistent citation of the relevant supporting literature. Experimentally, some of the most exciting conclusions are not supported by the data provided, this is particularly true for conclusions regarding functions of the microtubule domes with respect to sexual differentiation, Balbiani body formation and oocyte polarity. Although enthusiasm for the problem and the work is high there are several significant concerns with the study as presented including those mentioned above and detailed below.

© 2024. Published by The Company of Biologists under the terms of the Creative Commons Attribution License (https://creativecommons.org/licenses/by/4.0/).

Comments for the author

Major:
1) Summary statement: This manuscript identifies a novel microtubule structure leading to oocyte polarity.”
Based on the data provided, this is an exciting hypothesis worth testing but the data provided do not demonstrate that this is the case. The data provided support the conclusion that domes form earlier than previously described subcellular asymmetric structures like the Balbiani body. However, no functional evidence is provided demonstrating that lack of the dome structure leads to deficits in Bb formation or polarity, which would be required to conclude that these domes are required for polarity.
2) Further, although the data and analyses of wild type and foxl3 and dmrt1 mutants do support the conclusion that these structures are sexually dimorphic, based on the data provided it is not clear what regulates their dynamics (formation and stabilization in ovary or destabilization in testis). For example, the authors conclude that destabilization is downstream of Dmrt1, but it is not clear if this is direct or simply because the gonad remains an ovary or cannot adopt a testis fate. Based on the data provided, it is unclear how or if the domes contribute to sexual differentiation and fertility. Use of sex specific somatic gonad markers might guide analysis of cysts and interpretation of phenotypes in the various mutant contexts. In addition, in the experiments in Fig. 3, the cysts being compared among the different genotypes do not appear to be the same size, there appear to be different numbers of cells and in sizes among the genotypes.
Because these structures are dynamic, it seems important to compare cysts of the same size.
3) Interpretation of Dazl’s role is confounded by the requirement for Dazl in progression of germ cell development. It is unclear in the data provided which stage is being examined in the mutants. The stage is not indicated in the legend or on panels (Figure 2 E-F’) and the stage in the graph in panel 2G is listed as 0 dph.
What stage is 0dph? What stages of germ cell development are present in wild type and mutants at the stage being compared? Only a single time point 0 dph is shown for dazl mutants so it is not clear if the domes form and then are lost in dazl mutants or if they fail to form?
4) The model in Figure 5 might lead to confusion. Dazl is placed upstream of Meioc and commitment to gametogenesis and oocyte polarization is placed parallel and downstream of Dazl. It also implies that Dmrt1 is acting in oocytes to block polarization, but it most likely acts on the mitotic cysts or indeterminant
gonocytes that are becoming testis. It would be helpful to add these details or add a temporal timeline to the model.
5) There are inconsistencies between the literature cited and observations being referred to throughout the text. In some cases, reviews are cited. For example, after “movement along MTs”, the Elkouby 2017 paper cited is a review. Elkouby 2016 showed involvement of Microtubules and cilia but did not show transport. The primary literature demonstrating transport should be cited or the sentence revised. This and additional examples are detailed below in the “minor but important” section.
6) In Figure 1, the dramatic changes in acetylated tubulin abundance from type B SG to secondary SC is striking. Why such an increase from type -B SG to pachytene SC only to lose it again in secondary SC and then gain back in spermatids? Please also indicate how many individuals were examined and how many cysts showed these patterns?
7) In the model in Fig. 5, microtubules are drawn asymmetrically in the cartoon corresponding to pachytene SC, but in the image shown acetylated tubulin does not appear to be polarized. This should be reconciled.
8) Figure 4A: Are there really no mitochondria in zygotene cells?
9) “In this study, we identified a novel genetic pathway that regulate microtubule organization and lays the basis for oocyte polarity.” It is unclear which data support this statement.
10) “In addition, buc mRNA was co-localized with the MT dome in zygotene oocytes (Fig. 4C), suggesting that the MT dome frames the structure of Bb. Collectively, these data support the idea that the MT dome promotes oocyte development via organelle transfer and Bb formation”. Which data provide evidence for organelle transport? Are there markers, organelles, or mRNAs, that are not asymmetrically/co-localized at zygotene? Is this a function of the cytoplasmic space available or a specific event that occurs for some organelles and RNAs but not others?

© 2024. Published by The Company of Biologists under the terms of the Creative Commons Attribution License (https://creativecommons.org/licenses/by/4.0/).

11) “Interestingly, our results show that female-specific maintenance of this structure is independent of foxl3 but positive degradation in male by dmrt1 makes sexual difference of this structure.” This sentence is unclear as written and is potentially misleading. How can the authors be sure that Dmrt1 regulates microtubule destabilization as opposed to microtubule domes persisting due to failure of germ cells to take testis fate?
Are the domes destabilized in other contexts where germ cells cannot adopt testis fate?
12) The Elkouby 2016 paper is cited throughout but missing is citation of the 2022 Mytlis et al Science 2022 paper. The discussion would benefit from a comparison/contrast of the microtubule connections reported here to those reported in zebrafish in the Elkouby 2016 and Mytlis 2022 papers.

Minor but important:
1) Figure 3: Are the black and grey text meant to indicate levels of protein? If so, it would be helpful to add this to the legend.
2) Figure 3: On panels A-D, it would be helpful to outline the regions being magnified in the corresponding prime images.
3) Figure 4A: Please indicate whether the images projections or slices.
4) The manuscript would benefit from a careful proofreading to address the grammatical errors and potentially confusing sentences throughout the text. For example, from the abstract: “To acquire the sex-
specific characteristics of gametes, germ cells should initiate sex-specific genetic pathways after the sex determination.” And “Here we show presence of a novel pathway represented by a dome-shaped microtubule structure leading to the basis of oocyte polarity.” And “However, male-specific destabilization by dmrt1 is essential for the female specific maintenance of this structure.”
5) Line 54: The statement “Unlike mammals, the existence of sexually indifferent germline stem cells in both ovaries and testes has been shown in teleost fish, medaka (Oryzias latipes) (Nakamura et al., 2010;
Tanaka, 2016).” is potentially confusing as written – mammals do first develop sexually indifferent gonads that differentiate later, but germline stem cells are only found in testis.
6) “In Xenopus and zebrafish, the animal-vegetal axis of the egg is established in early oocytes by a structure called Balbiani body (Bb) (Elkouby, 2017; Jamieson-Lucy and Mullins, 68 2019).” Although it is true that the Balbiani body is established in oocytes, it has only been functionally demonstrated to establish the animal vegetal axis of the egg in zebrafish. The relevant primary literature should be cited here rather than or in addition to these reviews.
7) “These results indicate that Bb formation is an essential process for functional egg development
(Elkouby et al., 2016).” It is unclear why this paper is cited here since egg development was not examined in that study.
8) “In Drosophila, zebrafish, and mice, microtubule-mediated organelle transport among differentiating cyst cells is essential for Bb formation, which promotes oocyte development (Roper and Brown, 2004; Elkouby et al., 2016; Lei and Spradling, 2016; Niu and Spradling, 2022).” This sentence is potentially misleading. While microtubules have been implicated in each of these systems, transport from nurse cells has only been shown in Drosophila and mice. Further, microtubules were examined in earlier work from Xenopus, this work should be included as well.
9) “Interestingly, the timing of the loss of mitochondrial accumulation is consistent with that of loss of the microtubule extension that connects the MT domes (Fig. 1D).” This sentence is unclear. Are the authors suggesting that mitochondria are transferred among cyst cells, such that they are removed from some but not others?
10) “In zebrafish and mice, polarized distribution of the Bb component along microtubules is initially observed at around zygotene stage (Elkouby et al., 2016; Niu and Spradling, 2022). Similar to point 8, while microtubules have been implicated, polarized distribution of Bb components along microtubules was not demonstrated in the study cited.
11) “Buc is known to function in Bb assembly as an intrinsically disordered protein comprising a lattice-like meshwork (Bontems et al., 2009; Elkouby, 211 2017).” The Elkouby paper cited here is a review. Localization of endogenous Buc at zygotene and in the Balbiani body was reported in Heim et al Development 2014 and Elkouby Plos Bio 2016.
12) “Our results infer that germ cells initiate to implement a part of oocyte characteristics before the sex of the germ cell is determined.” This sentence is unclear as written. I think the authors saying that all germ cells initiate oocyte development regardless of whether they eventually become ovary or testis?

© 2024. Published by The Company of Biologists under the terms of the Creative Commons Attribution License (https://creativecommons.org/licenses/by/4.0/).

Reviewer 2

Advance summary and potential significance to field

Germ cells differentiate in sexually dimorphic gametes, the eggs or sperm.
Genetic cues will trigger the sex of the germ cells, mainly dmrt1bY in males and foxl3 in females in medaka. To be functional, germ cells will undergo several changes such as cell size, polarize macromolecules components as well as organelles. However, the underlying mechanism leading oocyte polarity remains to be uncovered especially in vertebrates. Medaka provides an exciting model to investigate oocyte polarity as the fate of the germ cells can be easily identify. Here, the authors report a novel microtubule structure present earlier than the sex is determined. The microtubule dome as it is named is in a sexually dimorphic fashion where its remains in oocyte but disappear in males but then restructure. The MT dome seems to function as a polarity marker.

Comments for the author

Strengths of the manuscript include the extensive and detailed characterization of the formation and maintenance of a newly discovered microtubule dome from PGCs to diplotene oocyte or sperm using various molecular and cellular tools and the finding that MT dome act as an early polarity marker in a undetermined sex germ cell. Most importantly, genetic analysis shows the implication of dmrt1 as a regulator of the microtubule structure and a passive regulation of foxl3. In XX females the examination of the MT dome has been elegantly shown as a precursor of the Balbiani body.
The weaknesses in the manuscript are largely presentational in nature and could be rather easily fixed by providing clarification and textual modifications. It will be nice to explain more in details in the discussion if there is any relationship between the fusome in Drosophila for example and the MT dome and if the MT dome is conserved in other species or it is medaka specific. Out of curiosity, even if in dazl mutant cells, the MT dome seems to be absent. This does not demonstrate that dazl is implicated in the formation of the MT structure. I will be eager to know the localization of dazl mRNA relative to the MT dome from PGC to diplotene oocyte. This is not a mandatory requirement from my side. These are clearly complex issues of high interest beyond the scope of the present study, which does provide useful data for the field. The manuscript is overall well written, well referenced (even if a more extensive body of work can be cited) and figures well presented. The title should be more representative of the findings by suggesting a novel microtubule structure leading to oocyte polarity on top of the microtubule network in both male and female germ cells.

Reviewer 3

Advance summary and potential significance to field

The authors claim to have discovered a novel microtubule based structure (the MT dome) that forms in early germ cells of medaka. It persists until late meiosis I prophase in oocytes, but is lost during early spermatogenesis. This structure appears to be destabilized in males via a dmrt1 pathway. The authors also suggests that the MT dome frames the structure of the Bilbiani body.

The work describes the organization of microtubules around nuclei of ooctyes and spermatocyte and how these structures change during gametogeneis. The findings are mainly descriptive and do not get at mechanistic events leading Bb formation. It would be significant to a larger audience if the discussion could compare and contrast the current findings with what is known in zebrafish.

Comments for the author

I am not convinced that the authors have adequately presented data to support these claims as several conclusions are made with no supporting data. These are detailed below. A major concern is that the authors have not ruled out another plausible model that the microtubules are part of the telomere bouquet that functions in meiosis to move telomeres around in the nucleus as part of the

© 2024. Published by The Company of Biologists under the terms of the Creative Commons Attribution License (https://creativecommons.org/licenses/by/4.0/).

homolog pairing process. This has been well characterized in zebrafish in the Elkouby lab. In this model, the MT structures associated with the dome are lost in both males and females and is replaced by these meiotic structures.

Another major concern is that the authors describe structures that are not seen in the figures or the proper probes were not used.

Examples include:

Figure 1: There is no evidence that the staining for gamma tubulin worked so conclusions about its location in the center of the dome cannot be stated.

Figure 4: There are no data supporting retention of Golgi occurs at zygotene.

The Golgi is shown only in one cell in one EM image that is likely pre-zygotene.

Figure 4C: I am not convinced that buc mRNA localization is an indication of where the Bb will form. Doesn't it just mean that buc is expressed at zygotene which precedes Bb formation?

For these reasons, I do not recommend publication of this paper in its current form.

Other comments:

Figure 1. In figure 1 C', there are some thin strands that are stained with AcTub. Is it possible these are cilium or consistent with what is observed in zebrafish? In figure 4b there seems to be a circular structure that could be a cilium?

Figure 1A: If the authors want to make the conclusion that the centrosome remains in the MT dome, they will need to show that it is present. It is not really obvious in any of the images

Line 124: To say that the dome is maintained in females and not males is not entirely supported by the data. The MT dome disappears and a network of cables form, starting from leptotene. The dome seems to disappear since MT structures in prophase do not have the characteristic hollow region seen in the early dome structure in the movies. I am wondering if these are analogous to the structures seen in type I OG or if they are MTs related to meiotic chromosome motion. In other words, what is seen in both zygotene/pachytene oocytes and spermatocytes is no longer considered the MT dome.

Supplementary Figure 1: Since this figure introduces the reader to the primary analysis, it seems important to be included in Figure 1.

Line 198: Why would it be expected that loss of mitochondria is consistent with loss of microtubule extension. How are these two events mechanistically related?

Consider replacing "consistent" with "concomitant" or "is correlated with.." so as not to imply causation.

Figure 3D": Please point the area in 3D that is enlarged in D' and D''. Also what is OVLAS? Why are there no MT structures? If these are cells that are entering prophase shouldn't MT persist?

Figure 4: If data support an alternate model that the MT dome does not persist as cells enter meiotic prophase I. There are no data supporting retention of Golgi occurs at zygotene. It is not clear in the EM image that the Golgi outlined in orange is at a MT dome since MTs cannot be seen. The text reports that the centrosome is "located at the center". Is it the two red dots? Please indicate on the EM image where they are for those not accustomed to looking at EM images.

Line 200: what does "circularly localized" mean

© 2024. Published by The Company of Biologists under the terms of the Creative Commons Attribution License (https://creativecommons.org/licenses/by/4.0/).

Line 208: The data in figure 4 do not support the conclusion that the MT dome acts as a precursor to the Bb. No Bb markers are stained. There is no evidence that the MTs are not the MTs to drive meiotic chromosome movement.

Second revision

Author response to reviewers' comments

Reviewer 1 Advance Summary and Potential Significance to Field:

Overall, this is an exciting observation, and the authors have attempted to determine the molecular hierarchy regulating sexual dimorphic maintenance of domes and potential roles of the domes in oocytes. However, the paper as written does not place the exciting findings in context and is difficult to understand in part due to a need for an introduction that better sets up the question(s) and places the work in the context of the field(s) – sex determination on one hand and oocyte patterning on the other. There are also potentially confusing or misleading sentences and grammatical errors that need to be addressed, as well as incomplete or inconsistent citation of the relevant supporting literature. Experimentally, some of the most exciting conclusions are not supported by the data provided, this is particularly true for conclusions regarding functions of the microtubule domes with respect to sexual differentiation, Balbiani body formation and oocyte polarity. Although enthusiasm for the problem and the work is high there are several significant concerns with the study as presented including those mentioned above and detailed below.

Response:

We appreciate the reviewer's comments of our work. We believe that the most original aspects of our study are that we provide evidence of sexual dimorphism of the microtubule structure in germ cells and insight into its regulatory mechanism. According to the reviewer's comments, we revised the Introduction, Results, and Discussion sections to clarify these points and to place our study in the context of sexual dimorphism (the revised portions are highlighted in yellow in the text). To respond to the reviewer's positive evaluation, we also added an experiment suggesting that the MT dome lays the foundation for the Balbiani body.

Reviewer 1 Comments for the Author:

Major:

1) *Summary statement: This manuscript identifies a novel microtubule structure leading to oocyte polarity." Based on the data provided, this is an exciting hypothesis worth testing but the data provided do not demonstrate that this is the case. The data provided support the conclusion that domes form earlier than previously described subcellular asymmetric structures like the Balbiani body. However, no functional evidence is provided demonstrating that lack of the dome structure leads to deficits in Bb formation or polarity, which would be required to conclude that these domes are required for polarity.*

Response:

The reviewer is correct, and we tested the possibility that the MT dome lays the basis for the Balbiani body. There is no method to disrupt microtubules specifically in medaka germ cells and there are no tool kits available that would allow us to perform the experiments in medaka. We therefore explored the way and found that nocodazole worked well. We treated medaka XX larvae with several concentrations of nocodazole for 10 hours and found that 10 μM nocodazole disrupted the MT dome structure in zygotene oocytes (revised Fig. S8). Loss of the MT dome resulted in dispersion of *bucky ball* mRNA in the cytoplasm of zygotene oocytes (revised Fig. 4D-E), suggesting that the MT dome is prerequisite for Balbiani body formation (lines 222-230). This point was also described in the Abstract (lines 44-45). We believe that these data support our argument and substantially improved the manuscript.

© 2024. Published by The Company of Biologists under the terms of the Creative Commons Attribution License (https://creativecommons.org/licenses/by/4.0/).

2) *Further, although the data and analyses of wild type and foxl3 and dmrt1 mutants do support the conclusion that these structures are sexually dimorphic, based on the data provided it is not clear what regulates their dynamics (formation and stabilization in ovary or destabilization in testis). For example, the authors conclude that destabilization is downstream of Dmrt1, but it is not clear if this is direct or simply because the gonad remains an ovary or cannot adopt a testis fate. Based on the data provided, it is unclear how or if the domes contribute to sexual differentiation and fertility. Use of sex specific somatic gonad markers might guide analysis of cysts and interpretation of phenotypes in the various mutant contexts.*

In addition, in the experiments in Fig. 3, the cysts being compared among the different genotypes do not appear to be the same size, there appear to be different numbers of cells and in sizes among the genotypes. Because these structures are dynamic, it seems important to compare cysts of the same size.

Response:

As pointed out by the reviewer, our genetic analyses do not reveal the detailed mechanism underlying the destabilization of the MT dome caused directly by *dmrt1* in testes. However, even the genetic basis of the mechanism has not been addressed previously. Our data clearly indicate the presence of a molecular pathway from somatic cells to germ cells that acts downstream of *dmrt1* to destabilize the MT dome in germ cells. We believe that this finding is entirely new.

If a germ cell-intrinsic masculinizer destabilizes the MT dome, the MT dome may not be detected in *dmrt1*$^{-/-}$; *foxl3*$^{-/-}$ XY gonads because *foxl3*$^{-/-}$ germ cells autonomously commit to spermatogenesis (Nishimura et al. 2015. Science). However, we detected the MT dome in double mutant germ cells, suggesting that the absence of *dmrt1* in somatic cells, but not the absence of *foxl3* in germ cells, is critical for the stability of the MT dome. To clarify this issue, we revised the manuscript as follows (lines 182–191):

"We hypothesized that somatic *dmrt1* is involved in the negative regulation of the MT dome in XY germ cells. To explore this possibility, the MT dome was examined in *dmrt1*/*foxl3*-double knockout (dKO) XY gonads, in which *dmrt1*-depleted somatic cells become feminized, whereas *foxl3*-depleted germ cells intrinsically commit to spermatogenesis. Consistently, spermatogenesis was observed in the double mutant ovaries due to the loss of *foxl3* in germ cells (tiny nuclei of sperm indicated by black arrowheads in Fig. 3C). We found that the MT dome remained stabilized in type-B spermatogonia of *dmrt1*/*foxl3*-dKO XY (Fig. 3C). This genetic analysis indicates that *dmrt1* in somatic cells regulates a downstream pathway that intercellularly promotes destabilization of the MT dome in wild- type XY germ cells (Fig. 3D)."

Regarding "it is unclear how or if the domes contribute to sexual differentiation and fertility", our *foxl3*$^{-/-}$ analysis shows that the MT dome is not required for completion of spermatogenesis. Regarding oogenesis, however, we showed that the MT dome is required for accumulation of *bucky ball* as described above. We added a discussion in the text (lines 261–284) supporting that the MT dome may positively contribute to oogenesis and affect oocyte polarization, female fertility, and embryogenesis.

Regarding the size of the cysts, we compared 8–16-cell cysts (after 3–4 mitotic divisions) among genotypes as suggested by the reviewer and revised the data shown in Fig. 3.

3) *Interpretation of Dazl's role is confounded by the requirement for Dazl in progression of germ cell development. It is unclear in the data provided which stage is being examined in the mutants. The stage is not indicated in the legend or on panels (Figure 2 E-F') and the stage in the graph in panel 2G is listed as 0 dph. What stage is 0dph? What stages of germ cell development are present in wild type and mutants at the stage being compared? Only a single time point 0 dph is shown for dazl mutants so it is not clear if the domes form and then are lost in dazl mutants or if they fail to form?*

Response:

In medaka, PGCs reach the primordial gonads and differentiate into gonocytes in stages 30–33 (2 days before hatching) (Nakamura et al. 2006. Dev Biol; Nishimura et al. 2014. Sex

© 2024. Published by The Company of Biologists under the terms of the Creative Commons Attribution License (https://creativecommons.org/licenses/by/4.0/).

Dev). Therefore, by stage 39 [0 day post- hatching (=0 dph)], all germ cells are already committed to gonocytes and divide mitotically (but meiosis has not yet begun).

Our previous study showed that *dazl*$^{-/-}$ germ cells remain in an undifferentiated PGC-like state. The mutant germ cells are morphologically similar to PGCs, which are characterized by smaller cell size and nucleoli than those in wild-type gonocytes, suggesting that *dazl*$^{-/-}$ germ cells fail to differentiate into gonocytes and likely lose competence for gametogenesis (Nishimura et al. 2018. PLoS Genet). Here, we showed that the MT dome is initially formed during the migrating stages of PGCs (approximately 80% of PGCs possess the MT dome in stage 22; Fig. 2G), and that *dazl*$^{-/-}$ PGC-like cells lose the MT dome in the gonad, suggesting that *dazl* is required for the persistence of the MT dome at the time of gonocyte development. We revised the manuscript (lines 152–163) and Fig. 2 legend to describe germ cell development and the phenotype of *dazl*$^{-/-}$ germ cells, which were previously analyzed in medaka. The subtitle of this paragraph was so revised to “The persistence of the MT dome requires *dazl* at the time of gonocyte development” as to be more accurate (line 148).

4) *The model in Figure 5 might lead to confusion. Dazl is placed upstream of Meioc and commitment to gametogenesis and oocyte polarization is placed parallel and downstream of Dazl. It also implies that Dmrt1 is acting in oocytes to block polarization, but it most likely acts on the mitotic cysts or indeterminant gonocytes that are becoming testis. It would be helpful to add these details or add a temporal timeline to the model.*

Response:

A previous study showed that *dazl* acts upstream of *meioC* and *foxl3* pathways, as transcripts for *meioC* and *foxl3* are not detected in *dazl*$^{-/-}$ germ cells at 0 dph (Nishimura et al. 2018. PLoS Genet). In addition, our data indicate that *dazl*, but not *meioC* or *foxl3*, is required for MT dome persistence during gonocyte development (Fig. 2E-G and Fig. 3B). To avoid confusion, we added a sentence explaining the genetic epistasis of *dazl* and related genes in lines 256–259.

Regarding the latter point, we revised Fig. 5B and its legend to show that somatic *dmrt1* intercellularly affects mitotic germ cells to suppress the MT dome pathway. In addition, we noted germ cell status above Fig. 5B.

5) *There are inconsistencies between the literature cited and observations being referred to throughout the text. In some cases, reviews are cited. For example, after “movement along MTs”, the Elkouby 2017 paper cited is a review. Elkouby 2016 showed involvement of Microtubules and cilia but did not show transport. The primary literature demonstrating transport should be cited or the sentence revised. This and additional examples are detailed below in the “minor but important” section.*

Response:

We thank the reviewer for pointing out the discrepancies. We carefully and thoroughly inspected the references and re-cited the appropriate ones.

6) *In Figure 1, the dramatic changes in acetylated tubulin abundance from type B SG to secondary SC is striking. Why such an increase from type -B SG to pachytene SC only to lose it again in secondary SC and then gain back in spermatids? Please also indicate how many individuals were examined and how many cysts showed these patterns?*

Response:

We are thinking that the increase in the abundance of acetylated tubulin in pachytene spermatocytes appears to be related to chromosome movement. During meiotic prophase-I, chromosomes are linked to microtubule motors to move along the nuclear membrane, which is implicated in homologous pairing and synapsis in various model organisms (Ding et al. 2007. Dev Cell; Shibuya et al. 2014. Nat Cell Biol; Sato et al. 2009, Cell; Labella et al. 2011. Dev Cell; Shimanuki et al. 1997. Mol Gen Genet). Therefore, it might be important to stabilize the microtubules at that specific stage. We discussed this point in the revised manuscript (lines 285–293).

Regarding the decrease in acetylated microtubules in secondary spermatocytes, we

© 2024. Published by The Company of Biologists under the terms of the Creative Commons Attribution License (https://creativecommons.org/licenses/by/4.0/).

speculate that any acetylated microtubules that are not required for cell division are depolymerized prior to meiotic metaphase-I. Actually, we observed mitotic germ cells at metaphase that always lose the MT dome (right figure).

NOTE: We have removed unpublished data that had been provided for the referees in confidence.

During spermiogenesis, acetylated microtubules are necessary for the formation of the sperm flagellum. Collectively, the dynamics of acetylated microtubules observed during spermatogenesis appear to be related to the roles of stable microtubules when they are required.
As suggested by the reviewer, we indicated the number of samples examined in the legend for Fig. 1 (10 dph XX: n=7; adult XY: n=4). The number of cysts examined for each differentiating stage of spermatogenesis is summarized in the table below (revised Table S1). All cysts at the same spermatogenic stages showed the same patterns, which was described in lines 120-121.

Table S1. The number of single germ cells or cysts observed in Figure 1J-N.

sample	# type-A SG	# type-B SG cyst	# pachytene SC cyst	# second. SC cyst	# ST cyst
1	10	10	10	10	0
2	10	10	10	6	3
3	10	10	9	9	9
4	10	10	6	6	6
total	40	40	35	31	18

7) *In the model in Fig. 5, microtubules are drawn asymmetrically in the cartoon corresponding to pachytene SC, but in the image shown acetylated tubulin does not appear to be polarized. This should be reconciled.*

Response:
We revised the Fig. 5 cartoon to reflect what was actually observed.

8) *Figure 4A: Are there really no mitochondria in zygotene cells?*

Response:
We meant to say that accumulation of mitochondria was not observed around the MT dome at the zygotene stage. Mitochondria were present and dispersed in the cytoplasm. We detected the CFP-mito signal in confocal images and observed mitochondria in TEM images (revised Fig. S4C"). Because mitochondrial dispersion made the CFP signal very dim in confocal images, we adjusted the contrast and indicated the ones we saw by arrows in Fig. 4A.

9) *"In this study, we identified a novel genetic pathway that regulate microtubule organization and lays the basis for oocyte polarity." It is unclear which data support this statement.*

Response:
We showed the presence of a MT dome pathway in germ cells that is affected by *dazl* (Fig. 2E–G) and in parallel with *foxl3* (Fig. 3B). This pathway is abolished by a signal from *dmrt1*-expressing somatic cells (Fig. 3C-D). In addition, as described above, we added new data indicating that the MT dome is required for *bucky ball* localization in oocytes, supporting our argument regarding oocyte polarization (Fig. 4D–E). Since we largely revised our manuscript according to the reviewer's suggestions, we believe that audience understands these points for the novel genetic pathway.

10)*"In addition, buc mRNA was co-localized with the MT dome in zygotene oocytes (Fig. 4C), suggesting that the MT dome frames the structure of Bb. Collectively, these data support the idea that the MT dome promotes oocyte development via organelle transfer and Bb formation".*

© 2024. Published by The Company of Biologists under the terms of the Creative Commons Attribution License (https://creativecommons.org/licenses/by/4.0/).

Which data provide evidence for organelle transport? Are there markers, organelles, or mRNAs, that are not asymmetrically/co-localized at zygotene? Is this a function of the cytoplasmic space available or a specific event that occurs for some organelles and RNAs but not others?

Response:

We showed the colocalization of mitochondria (CFP-mito) and the Golgi with the MT dome in mitotic germ cells (Fig. 4A, B). In addition, the timing of the loss of mitochondrial accumulation (zygotene, Fig. 4A) correlated with that of loss of the MT dome connections (zygotene, Fig. 1H). On the basis of these data, we revised our description in a more attenuated way that the MT dome might act as a base for transporting organelles from one cyst cell to the other (lines 260–269). We also deleted a sentence that might sound conclusively (lines 228–230). Because the focus of this manuscript was to demonstrate the presence of a sexually dimorphic structure of the microtubules and their genetic regulation during gametogenesis, we did not go into further mechanism of this point in this paper. This should be addressed in a future study.

Regarding the distribution of organelles and RNAs in zygotene oocytes, we found mitochondria that did not accumulate around the MT dome at the zygotene stage as mentioned above (Fig. 4A). In addition, VASA-containing germ granules were widely distributed in the cytoplasm of zygotene oocytes (right figure), suggesting that particular factors, such as *bucky ball*, may accumulate around the MT dome in zygotene oocytes.

NOTE: We have removed unpublished data that had been provided for the referees in confidence.

11)*"Interestingly, our results show that female-specific maintenance of this structure is independent of foxl3 but positive degradation in male by dmrt1 makes sexual difference of this structure." This sentence is unclear as written and is potentially misleading. How can the authors be sure that Dmrt1 regulates microtubule destabilization as opposed to microtubule domes persisting due to failure of germ cells to take testis fate? Are the domes destabilized in other contexts where germ cells cannot adopt testis fate?*

Response:

Dmrt1 is expressed in male-supporting cells. XY males of medaka *dmrt1* mutants turn into females with functional ovaries. Therefore, *dmrt1* is essential for gonad masculinization. On the other hand, *foxl3* is expressed in germ cells in the ovary but not in testis, and *foxl3*$^{-/-}$ XX germ cells undergo spermatogenesis in the ovary. Therefore, *foxl3* acts in a germ cell-autonomous manner. In *dmrt1*/*foxl3*-double knockout XY ovaries, *foxl3*-deficient germ cells autonomously commit to spermatogenesis and produce sperm, as observed in *foxl3*$^{-/-}$ XX ovaries (Nishimura et al. 2015. Science; lines 186-188). Therefore, we concluded that the MT dome was stabilized in the dKO ovaries because of the disruption of somatic *dmrt1*, but not because of the failure of germ cells to follow testis fate. To make this point clearer, we revised the sentence in the Discussion section as follows: "Genetic analyses showed that the male- specific disruption of the MT dome in germ cells was mediated by a pathway involving somatic *dmrt1*, but not germ cell *foxl3*" (lines 296–298).

12)*The Elkouby 2016 paper is cited throughout but missing is citation of the 2022 Mytlis et al Science 2022 paper. The discussion would benefit from a comparison/contrast of the microtubule connections reported here to those reported in zebrafish in the Elkouby 2016 and Mytlis 2022 papers.*

Response:

We thank the reviewer for pointing this out. We discussed the relationship between the cilium in zebrafish and the MT dome in medaka in the Discussion section (lines 285–293) and cited the paper (Elkouby et al. 2022. Science) throughout the manuscript as required.

Minor but important:

1) *Figure 3: Are the black and grey text meant to indicate levels of protein? If so, it would be helpful to add this to the legend.*

Response:

© 2024. Published by The Company of Biologists under the terms of the Creative Commons Attribution License (https://creativecommons.org/licenses/by/4.0/).

We thank the reviewer for the suggestion. We added the meaning of the colored letters to the legend.

2) *Figure 3: On panels A-D, it would be helpful to outline the regions being magnified in the corresponding prime images.*

Response:

We revised Fig. 3A–D and added rectangles to indicate the regions magnified in A'–D', A"-D".

3) *Figure 4A: Please indicate whether the images projections or slices.*

Response:

We indicated that the images are single-optical slices in the corresponding legend.

4) *The manuscript would benefit from a careful proofreading to address the grammatical errors and potentially confusing sentences throughout the text. For example, from the abstract: "To acquire the sex-specific characteristics of gametes, germ cells should initiate sex-specific genetic pathways after the sex determination." And "Here we show presence of a novel pathway represented by a dome-shaped microtubule structure leading to the basis of oocyte polarity." And* "However, male- *specific destabilization by dmrt1 is essential for the female specific maintenance of this structure."*

Response:

We thank the reviewer for this comment. The entire text was edited by a native English speaker. The sentences mentioned by the reviewer were revised as follows:

> *"To acquire the sex-specific characteristics of gametes, germ cells should initiate sex-specific genetic pathways after the sex determination."*

We deleted this sentence and revised the structure of the Abstract.

> *"Here we show presence of a novel pathway represented by a dome-shaped microtubule structure leading to the basis of oocyte polarity."*

We revised the sentence to "Here, we identified a novel pathway involving a dome-shaped microtubule structure that may lay the basis of oocyte polarity." (lines 38-40)

> *"However, male-specific destabilization by dmrt1 is essential for the female specific maintenance of this structure."*

We revised the sentence to "This structure was first established in primordial germ cells in both sexes, but was maintained only during oogenesis and destabilized in differentiating spermatogonia under the influence of Sertoli cells expressing *dmrt1*." (lines 40-42)

5) *Line 54: The statement "Unlike mammals, the existence of sexually indifferent germline stem cells in both ovaries and testes has been shown in teleost fish, medaka (Oryzias latipes) (Nakamura et al., 2010; Tanaka, 2016)." Is potentially confusing as written – mammals do first develop sexually indifferent gonads that differentiate later, but germline stem cells are only found in testis.*

Response:

We thank the reviewer for the comment. We removed "Unlike mammals" and revised the entire paragraph on lines 59-72

6) *"In Xenopus and zebrafish, the animal-vegetal axis of the egg is established in early oocytes by a structure called Balbiani body (Bb) (Elkouby, 2017; Jamieson-Lucy and Mullins, 68 2019)." Although it is true that the Balbiani body is established in oocytes, it has only been functionally demonstrated to establish the animal vegetal axis of the egg in zebrafish. The relevant primary literature should be cited here rather than or in addition to these reviews.*

Response:

We thank the reviewer for the comment. The primary literature was cited in our revised

© 2024. Published by The Company of Biologists under the terms of the Creative Commons Attribution License (https://creativecommons.org/licenses/by/4.0/).

manuscript. Because we changed the structure of the Introduction, the relevant sentence was moved to the Results and Discussion sections (lines 211–216, 275–284).

7) *“These results indicate that Bb formation is an essential process for functional egg development (Elkouby et al., 2016).” It is unclear why this paper is cited here since egg development was not examined in that study.*

Response:

The reviewer is correct. The relevant text and citations were revised as noted in a previous response above.

8) *“In Drosophila, zebrafish, and mice, microtubule-mediated organelle transport among differentiating cyst cells is essential for Bb formation, which promotes oocyte development (Roper and Brown, 2004; Elkouby et al., 2016; Lei and Spradling, 2016; Niu and Spradling, 2022).” This sentence is potentially misleading. While microtubules have been implicated in each of these systems, transport from nurse cells has only been shown in Drosophila and mice. Further, microtubules were examined in earlier work from Xenopus, this work should be included as well.*

Response:

We thank the reviewer for this comment. We removed “zebrafish” and the corresponding reference paper (Elkouby et al., 2016) from the sentence (lines 196-199). However, we could not find clear work showing the relation between microtubules and Bb formation in *Xenopus*.

9) *“Interestingly, the timing of the loss of mitochondrial accumulation is consistent with that of loss of the microtubule extension that connects the MT domes (Fig. 1D).” This sentence is unclear. Are the authors suggesting that mitochondria are transferred among cyst cells, such that they are removed from some but not others?*

Response:

To clarify what we wanted to say, we added the following discussion: “First, we showed that the MT dome in medaka is positioned in an organelle-enriched area and extends microtubule bundles toward adjacent germ cells in a cyst via intercellular bridges (Fig. 1F–G, Fig. 4A). This association disappears as intercellular connections are lost during meiotic prophase I. This implies that the MT dome in medaka functions as a center for microtubule extension connecting different cystic germ cells and is consequently involved in organelle and cytoplasmic transfer. This is consistent with previous studies in *Drosophila* and mice showing microtubule-dependent organelle transport among ovarian cystic germ cells via intercellular bridges (Roper and Brown, 2004; Lei and Spradling, 2016; Niu and Spradling, 2022).” (lines 261–269)

10) *“In zebrafish and mice, polarized distribution of the Bb component along microtubules is initially observed at around zygotene stage (Elkouby et al., 2016; Niu and Spradling, 2022). Similar to point 8, while microtubules have been implicated, polarized distribution of Bb components along microtubules was not demonstrated in the study cited.*

Response:

We thank the reviewer for this comment. In this context, we focused on the asymmetric distribution of the Bb components, especially *bucky ball*. Thus, we cited only a zebrafish paper (Elkouby et al., 2016), in which the localization of bucky ball in oocytes was observed as early as in the zygotene stage (lines 219–221).

11) *“Buc is known to function in Bb assembly as an intrinsically disordered protein comprising a lattice-like meshwork (Bontems et al., 2009; Elkouby, 211 2017).” The Elkouby paper cited here is a review. Localization of endogenous Buc at zygotene and in the Balbiani body was reported in Heim et al Development 2014 and Elkouby Plos Bio 2016.*

Response:

We thank the reviewer for this comment. We corrected the references and revised the sentence as follows. “Bucky ball (Buc) proteins and transcripts localize to and function in Bb assembly in zebrafish, stabilizing ribonucleoproteins to the Bb (Marlow and Mullins, 2008; Bontems et al., 2009; Heim et al., 2014; Elkouby et al., 2016).” (lines 214-216)

© 2024. Published by The Company of Biologists under the terms of the Creative Commons Attribution License (https://creativecommons.org/licenses/by/4.0/).

12) *"Our results infer that germ cells initiate to implement a part of oocyte characteristics before the sex of the germ cell is determined." This sentence is unclear as written. I think the authors saying that all germ cells initiate oocyte development regardless of whether they eventually become ovary or testis?*

Response:

The reviewer's interpretation is correct. To make the sentence clearer, we revised it as follows. "The present results suggest that germ cells are intrinsically directed to become oocytes, as suggested by the acquisition of oocyte-specific polarity from their primordial state." (lines 300–302)

Reviewer 2 Comments for the Author:

Strengths of the manuscript include the extensive and detailed characterization of the formation and maintenance of a newly discovered microtubule dome from PGCs to diplotene oocyte or sperm using various molecular and cellular tools and the finding that MT dome act as an early polarity marker in a undetermined sex germ cell. Most importantly, genetic analysis shows the implication of dmrt1 as a regulator of the microtubule structure and a passive regulation of foxl3. In XX females the examination of the MT dome has been elegantly shown as a precursor of the Balbiani body. The weaknesses in the manuscript are largely presentational in nature and could be rather easily fixed by providing clarification and textual modifications. It will be nice to explain more in details in the discussion if there is any relationship between the fusome in Drosophila for example and the MT dome and if the MT dome is conserved in other species or it is medaka specific. Out of curiosity, even if in dazl mutant cells, the MT dome seems to be absent. This does not demonstrate that dazl is implicated in the formation of the MT structure. I will be eager to know the localization of dazl mRNA relative to the MT dome from PGC to diplotene oocyte. This is not a mandatory requirement from my side. These are clearly complex issues of high interest beyond the scope of the present study, which does provide useful data for the field. The manuscript is overall well written, well referenced (even if a more extensive body of work can be cited) and figures well presented. The title should be more representative of the findings by suggesting a novel microtubule structure leading to oocyte polarity on top of the microtubule network in both male and female germ cells.

Response:

We appreciate the reviewer's positive evaluation of our work.

> *It will be nice to explain more in details in the discussion if there is any relationship between the fusome in Drosophila for example and the MT dome and if the MT dome is conserved in other species or it is medaka specific.*

Response:

We appreciate the important suggestions from the reviewer. As commented, we revised the Discussion section and described three possible functions of the MT dome: organelle transport, Bb formation, and chromosome movement by comparison with other microtubule structures reported in other organisms, such as *Drosophila*, mouse, and zebrafish (lines 260–293).

Regarding the fusome, it binds to the "Shot" protein, which interacts with microtubules to support the transportation of cytoplasmic components from nurse cells to pro-oocytes (Roper et al. 2004. Curr Biol). We speculate that the MT dome in medaka may play a similar role in organelle transport for oocyte development.

The MT dome-like structure has not been reported in any other organisms. However, a similar asymmetric distribution of microtubules was reported in oocytes of *Drosophila* (Theurkauf et al. 1993. Development; Roper and Brown, 2004. Curr Biol), zebrafish (Elkouby et al. 2016. PLoS Biol; Mytlis et al. 2022. Science), and mouse (Lei and Spradling, 2016. Science; Niu and Spradling, 2022. Cell). It is interesting that microtubules may show functional homology and accumulate some components of the Balbiani bodies in the oocyte to establish the polarity.

> *Out of curiosity, even if in dazl mutant cells, the MT dome seems to be absent. This does not demonstrate that dazl is implicated in the formation of the MT structure. I will be eager to know*

© 2024. Published by The Company of Biologists under the terms of the Creative Commons Attribution License (https://creativecommons.org/licenses/by/4.0/).

the localization of dazl *mRNA relative to the MT dome from PGC to diplotene oocyte. This is not a mandatory requirement from my side.*

Response:

Thank you for this comment. Our previous study suggested that *dazl* is required for the differentiation of PGCs into gonocytes in medaka (Nishimura et al. 2018. PLoS Genet). In *dazl*$^{-/-}$ gonads, germ cells that reach the gonad remain in a PGC-like state characterized by a smaller cell size and nucleolus. In this study, *dazl*$^{-/-}$ germ cells at 0 dph exhibited the same PGC-like state, as well as loss of the MT dome (Fig. 2E, G). Because the MT dome is initially formed in PGCs, we speculate that *dazl*$^{-/-}$ PGCs formed MT domes but failed to maintain them during differentiation into gonocytes.

Reviewer 3 Comments for the Author:

A major concern is that the authors have not ruled out another plausible model that the microtubules are part of the telomere bouquet that functions in meiosis to move telomeres around in the nucleus as part of the homolog pairing process. This has been well characterized in zebrafish in the Elkouby lab. In this model, the MT structures associated with the dome are lost in both males and females and is replaced by these meiotic structures.

Another major concern is that the authors describe structures that are not seen in the figures or the proper probes were not used.

Examples include:

Figure 1*: There is no evidence that the staining for gamma tubulin worked so conclusions about its location in the center of the dome cannot be stated.*

Response:

A commercially available rabbit antibody (GeneTex, GTX113286) for γ-tubulin was employed which guarantees the reactivity for vertebrates from zebrafish to human and has widely been used in many other vertebrates. In fact, in our experiments, the γ-tubulin antibody specifically detected centrosomes that were obviously located in the MT dome, as shown in revised Fig. 1A–D and Fig. S1. In addition, we often observed the signals in spindle poles in mitotically dividing cells (right figure). We think there is no doubt regarding the specificity of the antibody.

Figure 4*: There are no data supporting retention of Golgi occurs at zygotene. The Golgi is shown only in one cell in one EM image that is likely pre-zygotene.*

NOTE: We have removed unpublished data that had been provided for the referees in confidence.

Response:

To address the reviewer's concern, Fig. S4C was provided as another TEM image of zygotene oocyte in the revised manuscript in addition to the original TEM images of type-I oogonia (Fig. 4B). In zygotene oocytes, synapsed chromosomes are evident in the nucleus (black arrowheads in Fig. S4C). These images show the localization of Golgi stacks around the centrosomes.

Figure 4C*: I am not convinced that buc mRNA localization is an indication of where the Bb will form. Doesn't it just mean that buc is expressed at zygotene, which precedes Bb formation?*

Response:

buc mRNA has been shown to localize to the Bb in zebrafish oocytes (Heim et al., 2014, Development). In the zebrafish manuscript, the mRNA localization is shown to be important for promoting the accumulation of other Bb components. Similarly, in medaka, we observed the specific localization of *bucky ball* mRNA to the region where the MT dome is located (Fig. 4C and newly provided Fig. 4D–E). If it only reflects the expression of the *buc* gene, the mRNA should be found anywhere in the cytoplasm, but it was localized specifically around the MT dome.

Other comments:

© 2024. Published by The Company of Biologists under the terms of the Creative Commons Attribution License (https://creativecommons.org/licenses/by/4.0/).

***Figure 1**. In figure 1 C', there are some thin strands that are stained with AcTub. Is it possible these are cilium or consistent with what is observed in zebrafish? In figure 4b there seems to be a circular structure that could be a cilium?*

Response:

Thank you for the comment. We also suspected that the thin microtubule cables are the cilia that have been described in zebrafish meiocytes (Mytlis et al. 2022. Science). Therefore, before our initial submission, we carefully observed the structure and tried to detect the cilia many times. However, the thin strand that the reviewer pointed out is not the cilium. We found the cables to be always connected to the MT domes in neighboring cyst cells through intercellular bridges (Movie 2), which is consecutively observed from mitotically dividing germ cells (revised Fig.1F, F') to leptotene germ cells (revised Fig.1G, G'). In Fig. 4B, we do not observe "a circular structure" as mentioned by the reviewer. Our careful search for ciliated structures in the TEM images did not reveal them.

***Figure 1A**: If the authors want to make the conclusion that the centrosome remains in the MT dome, they will need to show that it is present. It is not really obvious in any of the images*

Response:

As mentioned above, as well as in the manuscript (lines 92-94), we provided a clear image of centrosomal localization at the base of the MT dome including the 3D reconstruction in revised Fig. S1. I believe that this image will convince the reviewer.

***Line 124**: To say that the dome is maintained in females and not males is not entirely supported by the data. The MT dome disappears and a network of cables form, starting from leptotene. The dome seems to disappear since MT structures in prophase do not have the characteristic hollow region seen in the early dome structure in the movies. I am wondering if these are analogous to the structures seen in type I OG or if they are MTs related to meiotic chromosome motion. In other words, what is seen in both zygotene/pachytene oocytes and spermatocytes is no longer considered the MT dome.*

Response:

The cable structure that connects the MT domes is obvious in cystic (or type-II) oogonia, as shown in revised Fig. 1F and Movie 2. The MT domes with a hollow inside are consecutively observed from type-I oogonia to zygotene oocytes (Movies 1–4), and they always possess a centrosome inside, suggesting that the MT dome is persistently maintained in female germ cells. In primary spermatocytes, we did not observe such a conspicuous dome-like structure with a hollow inside, although we found that microtubules become abundant when they initiate meiosis (Fig. 1L) which is apparently a different structure from the MT dome. This may represent the movement of meiotic chromosomes along the nuclear membrane as described in many organisms. Our data do not rule out the possibility that the MT dome in meiotic oocytes is involved in chromosomal movement as occurs in zebrafish. Rather, we speculate that the MT dome in female germ cells may play several roles during oogenesis, including organelle transport, Bb formation, and meiotic chromosomal movement. We revised the manuscript and discussed the possible role of the MT dome in chromosome movement in lines 285–293.

***Supplementary Figure 1**: Since this figure introduces the reader to the primary analysis, it seems important to be included in Figure 1.*

Response:

We thank the reviewer for this suggestion. Original Fig. S1 was moved to revised Fig. 1A–D.

***Line 198**: Why would it be expected that loss of mitochondria is consistent with loss of microtubule extension. How are these two events mechanistically related? Consider replacing "consistent" with "concomitant" or "is correlated with.." so as not to imply causation.*

Response:

We thank the reviewer for this suggestion. As suggested, the corresponding sentence was revised as follows. "The timing of the loss of mitochondrial accumulation correlated with

© 2024. Published by The Company of Biologists under the terms of the Creative Commons Attribution License (https://creativecommons.org/licenses/by/4.0/).

the loss of the microtubule extension from the MT domes" (lines 206-207).

Figure 3D"*: Please point the area in 3D that is enlarged in D' and D''. Also, what is OVLAS? Why are there no MT structures? If these are cells that are entering prophase shouldn't MT persist?*

Response:

We indicated the enlarged area in Fig. 3D by white boxes. OLVAS (Oryzias latipes VASA) is alias for VASA. The notation was standardized to VASA throughout the manuscript.

The cystic germ cells indicated in Fig. 3D'-D" are differentiating spermatogonia (can be identified by distinct nucleoli), and therefore they have not entered meiosis yet. As shown in the figure on the right hand (modified from Fig. 3D), meiotic primary spermatocytes (can be easily recognized by an ambiguous nucleolus with more condensed chromosomes) have abundant microtubules (green signals). Because we intended to show the genetic pathway involved in the destabilization of the MT dome by *dmrt1* in differentiating spermatogonia, the differentiating spermatogonia were highlighted in Fig. 3D in the text.

NOTE: We have removed unpublished data that had been provided for the referees in confidence.

Figure 4*: If data support an alternate model that the MT dome does not persist as cells enter meiotic prophase I. There are no data supporting retention of Golgi occurs at zygotene. It is not clear in the EM image that the Golgi outlined in orange is at a MT dome since MTs cannot be seen. The text reports that the centrosome is "located at the center". Is it the two red dots? Please indicate on the EM image where they are for those not accustomed to looking at EM images.*

Response:

Immunostaining for acetylated α-tubulin and γ-tubulin showed that the MT dome persists until the zygotene stage of meiotic prophase I. However, the microtubule cables that connect the MT domes in neighboring germ cells appear to be lost by the zygotene stage. The location of the MT dome in TEM images was estimated from the position of centrosomes, which are indicated by red asterisks in revised Fig. 4B and Fig. S3C'.

Line 200*: what does "circularly localized" mean*

Response:

We meant to say that Golgi stacks were arranged in a circular manner around the centrosomes. We corrected the sentence to "Transmission electron micrographs (TEM) of type-I oogonia and zygotene oocytes showed that Golgi stacks were arranged in a circular pattern around centrosomes, at the location where the MT dome is supposed to form (Fig. 4B, Fig. S4)." (lines 201-204).

Line 208*: The data in figure 4 do not support the conclusion that the MT dome acts as a precursor to the Bb. No Bb markers are stained. There is no evidence that the MTs are not the MTs to drive meiotic chromosome movement.*

Response:

We provided new data showing that loss of the MT dome resulted in dispersion of *bucky ball* mRNA (revised Fig. 4D- E). *Bucky ball* mRNA has been shown to localize to the Bb in zebrafish (Heim et al. 2014. Development) as well as in medaka (Fig. S7). In this study, we do not exclude the possibility that the MT dome is involved in chromosome movement during meiotic prophase I. The focus of this manuscript was to show sexual differences in microtubule structures and their regulation in germ cells, which we believe is a new finding. Understanding the relationship between chromosome dynamics and the microtubule network in medaka is of great interest for future research.

Third decision letter

© 2024. Published by The Company of Biologists under the terms of the Creative Commons Attribution License (https://creativecommons.org/licenses/by/4.0/).

MS ID#: DEVELOP/2023/201840

MS TITLE: Sexually dimorphic dynamics of the microtubule network in medaka (*Oryzias latipes*) germ cells

AUTHORS: Mariko Kikuchi, Miyo Yoshimoto, Tokiro Ishikawa, Yuto Kanda, Kazutoshi Mori, Toshiya Nishimura, and Minoru Tanaka

I have now received all the referees reports on the above manuscript, and have reached a decision. The referees' comments are appended below, or you can access them online: please go to BenchPressand click on the 'Manuscripts with Decisions' queue in the Author Area.

The overall evaluation is positive and we would like to publish a revised manuscript in Development. Reviewer 1 makes some suggestions to further improve the readability of the manuscript. I do not anticipate sending the manuscript back to the reviewer, however, I ask that you please attend to all of the reviewers' comments in your revised manuscript and detail them in your point-by-point response. If you do not agree with any of their criticisms or suggestions explain clearly why this is so.

Reviewer 1

Advance summary and potential significance to field

In many animals, including mammals, gonad development begins with sexually indifferent early germ cells and somatic gonad precursors that form a sexually indifferent gonad that later differentiates as ovary or testis. The mechanisms that regulate specification of the early germline and later sexual differentiation of the gonad vary widely but involve factors, many of which are conserved, that trigger molecular and cellular events that regulate gonad differentiation as ovary or testis. Understanding the earliest cellular and molecular events in sexual differentiation of the gonads is an interesting and significant problem. In this revised manuscript from Kikuchi and colleagues, the authors examined microtubule organization in developing medaka germ cells from embryonic stages through early gonad formation and differentiation as ovary or testis and identified a sexual dimorphism in the pattern of microtubule organization in mitotic germ cells. They identified a novel microtubule asymmetry in mitotic cysts of sexually undifferentiated gonads.

Specifically, the presence of dome structures that connect cells within cysts in ovaries, but not in differentiated testis. They make use of previously established mutant lines to explore the relationship between these microtubule domes and connections and early germline development (dazl mutants) and sex determination (foxl3 and dmrt1 mutants). This analyses confirms the ovary and testis specific patterns and presence of microtubule domes. In terms of ovary development, the beautiful images show asymmetric microtubule structures at stages of development before asymmetry has been reported thus far. Using gamma tubulin as a marker of centrosomes, they show that microtubule domes form around these microtubule organizing centers at stages before earlier oocyte asymmetries, the Balbiani body specifically, form. To examine the relationship between microtubule domes and Balbiani body formation, they examine acetylated microtubules and the localization of transcripts encoding Medaka homologs of a regulator of Balbiani body development in zebrafish, bucky ball. Because domes form first and buc later localizes proximal to the centrosome, the authors conclude that microtubule domes are precursors to the Balbiani bodies and later polarity that forms in oocytes. In this revised manuscript they tested this hypothesis using microtubule disrupting drugs. The new data show that the microtubule poisons disrupt asymmetric accumulation of bucl RNA, a regulator of Bb development. Based on this observation they conclude that the dome is the precursor of the Bb.

This is an exciting finding and the authors have significantly revised and improved the manuscript and have addressed most of the concerns raised previously. However, there are a few mostly minor points that remain to be addressed. As detailed below, these are minor writing edits as well as including alternative or qualifying statements for some of the conclusions. Overall enthusiasm for this work is high.

© 2024. Published by The Company of Biologists under the terms of the Creative Commons Attribution License (https://creativecommons.org/licenses/by/4.0/).

Comments for the author

Minor but important
1) Figure 4 Panel D: The nocodozole treated cell nuclei appear to be in a central position. Was DAPI or
Hoechst used to control for stage? This result seems to suggest that the MTs are important for asymmetric position of the nuclei, which could be prerequisite for the cytoplasmic asymmetries. How the cells were staged should be clarified and this possibility should be discussed.
Minor
1)Line 30 of summary: “is prepared” consider instead “forms” or “is present”.
2) Line 39: “be” or “establish” rather than “lay”.
3) Line 41: “was” is needed between “and destabilized”
4) Line 45: “dispersal” rather than “dispersion”.
5) Line 45: “suggesting the structure is prerequisite for Balbiani body”. Either qualify by stating it “may be prerequisite” or change to “localizing this Balbiani body factor” since only one marker was examined.
6) Line 54: “within” rather than “into” otherwise readers might think the germ cells become somatic gonad.
7) Line 65 and line 73: “the” is not needed before examination.
8) Line 73: “with” or “in the context of” rather than “to the”.
9) Line 79: “and is getting replaced with Balbiani body (Bb) in formation at the zygotene stage”. Consider instead “and is replaced with the Balbiani body, which forms at zygotene stage..”
10) Line 215: Rather than “act as a base for” consider “act to facilitate organelle transport..”
11) Line 236: “which lays the foundation for Bb formation”. Consider instead “a key regulator of Bb formation”.
12) Line 284: “suggests that the Bb replaces the..” consider instead “is important for recruiting this Bb regulator and possibly for Bb assembly.”
13) Line 286: “it thus sets a frame” . Consider instead “accordingly would set the foundation for..”

<u>Reviewer 2</u>

Advance summary and potential significance to field

Kikuchi et al have done a terrific job at reshaping the manuscript to a more comprehensive one by presenting in a clearer way the sex differences during germ cell ontology in medaka. The focus of the paper is now understandable. My concerns have been addressed even if the bibliography has been directed toward specific papers and my curiosity still want to see dazl mRNA localization from the MT dome formation to the Balbiani body as dazl is required for the persistence of the dome. Overall, the findings and the manuscript are of great interest for the field.

Comments for the author

No additional comments

Third revision

<u>Author response to reviewers' comments</u>

Reviewer 1 Advance Summary and Potential Significance to Field:
In many animals, including mammals, gonad development begins with sexually indifferent early germ cells and somatic gonad precursors that form a sexually indifferent gonad that later differentiates as ovary or testis. The mechanisms that regulate specification of the early germline and later sexual differentiation of the gonad vary widely but involve factors, many of which are conserved, that trigger molecular and cellular events that regulate gonad differentiation as ovary

© 2024. Published by The Company of Biologists under the terms of the Creative Commons Attribution License (https://creativecommons.org/licenses/by/4.0/).

or testis. Understanding the earliest cellular and molecular events in sexual differentiation of the gonads is an interesting and significant problem. In this revised manuscript from Kikuchi and colleagues, the authors examined microtubule organization in developing medaka germ cells from embryonic stages through early gonad formation and differentiation as ovary or testis and identified a sexual dimorphism in the pattern of microtubule organization in mitotic germ cells. They identified a novel microtubule asymmetry in mitotic cysts of sexually undifferentiated gonads. Specifically, the presence of dome structures that connect cells within cysts in ovaries, but not in differentiated testis. They make use of previously established mutant lines to explore the relationship between these microtubule domes and connections and early germline development (dazl mutants) and sex determination (foxl3 and dmrt1 mutants). This analyses confirms the ovary and testis specific patterns and presence of microtubule domes. In terms of ovary development, the beautiful images show asymmetric microtubule structures at stages of development before asymmetry has been reported thus far. Using gamma tubulin as a marker of centrosomes, they show that microtubule domes form around these microtubule organizing centers at stages before earlier oocyte asymmetries, the Balbiani body specifically, form. To examine the relationship between microtubule domes and Balbiani body formation, they examine acetylated microtubules and the localization of transcripts encoding Medaka homologs of a regulator of Balbiani body development in zebrafish, bucky ball. Because domes form first and buc later localizes proximal to the centrosome, the authors conclude that microtubule domes are precursors to the Balbiani bodies and later polarity that forms in oocytes. In this revised manuscript they tested this hypothesis using microtubule disrupting drugs. The new data show that the microtubule poisons disrupt asymmetric accumulation of bucl RNA, a regulator of Bb development. Based on this observation they conclude that the dome is the precursor of the Bb.

This is an exciting finding and the authors have significantly revised and improved the manuscript and have addressed most of the concerns raised previously. However, there are a few mostly minor points that remain to be addressed. As detailed below, these are minor writing edits as well as including alternative or qualifying statements for some of the conclusions. Overall enthusiasm for this work is high.

Reviewer 1 Comments for the Author:
Minor but important:
1) *Figure 4 Panel D: The nocodozole treated cell nuclei appear to be in a central position. Was DAPI or Hoechst used to control for stage? This result seems to suggest that the MTs are important for asymmetric position of the nuclei, which could be prerequisite for the cytoplasmic asymmetries. How the cells were staged should be clarified and this possibility should be discussed.*

Response:

We identified zygotene oocytes by DAPI-stained condensed chromosomes forming a bouquet structure and by localization of nucleoli near the nuclear membrane (as shown in Fig. S8I). We added the method for staging zygotene oocytes to the revised manuscript in lines 413-414.

As the reviewer suggests, disruption of the MT dome also caused localization of the nucleus around the center of the cells (Fig. 4D), which is of clear difference from that of wild-type, where the nucleus is positioned asymmetrically in cytoplasm. We revised the manuscript as follows (underlined) for the interesting perspective in lines Discussion section (227-279).

"Second, our observation that disruption of the MT dome by nocodazole results in dispersion of *bucl* mRNA in zygotene oocytes suggests that the MT dome is important for recruiting this Bb regulator and possibly for Bb assembly. Disruption of the MT dome also resulted in localization of the nucleus to the center of the cells, in contrast to the wild-type where the nucleus is asymmetrically positioned in cytoplasm (Fig. 4D). Thus, the MT dome may be a prerequisite for the acquisition of oocyte polarity by predetermining the location, and it accordingly would set the foundation for the Bb."

Minor
1) *Line 30 of summary: "is prepared" consider instead "forms" or "is present".*

Response:

© 2024. Published by The Company of Biologists under the terms of the Creative Commons Attribution License (https://creativecommons.org/licenses/by/4.0/).

We revised “is prepared” to “is present” (line 30).

2) *Line 39: “be” or “establish” rather than “lay”.*

Response:
We revised “lay” to “be” (line 39).

3) *Line 41: “was” is needed between “and destabilized”*

Response:
We added “was” in the appropriate position (line 41).

4) *Line 45: “dispersal” rather than “dispersion”.*

Response:
We revised “dispersion” to “dispersal” (line 45).

5) *Line 45: “suggesting the structure is prerequisite for Balbiani body”. Either qualify by stating it “may be prerequisite” or change to “localizing this Balbiani body factor” since only one marker was examined.*

Response:
We revised the sentence to “In addition, disruption of the microtubule dome caused dispersal of *bucky ball* RNA, suggesting the structure may be prerequisite for Balbiani body.” (lines 44-46).

6) *Line 54: “within” rather than “into” otherwise readers might think the germ cells become somatic gonad.*
Response:
We revised “into” to “within” (line 55).

7) *Line 65 and line 73: “the” is not needed before examination.*

Response:
We deleted “the” from the relevant sentences (line 66 and line 72).

8) *Line 73: “with” or “in the context of” rather than “to the”.*

Response:
We revised “to the” to “with” (line 72).

9) *Line 79: “and is getting replaced with Balbiani body (Bb) in formation at the zygotene stage”. Consider instead “and is replaced with the Balbiani body, which forms at zygotene stage..”*

Response:
We revised the relevant text as suggested by the reviewer (line 77).

10) *Line 215: Rather than “act as a base for” consider “act to facilitate organelle transport..”*

Response:
We revised “act as a base for” to “act to facilitate” (line 209).

11) *Line 236: “which lays the foundation for Bb formation”. Consider instead “a key regulator of Bb formation”.*

Response:

© 2024. Published by The Company of Biologists under the terms of the Creative Commons Attribution License (https://creativecommons.org/licenses/by/4.0/).

We revised "which lays the foundation for Bb formation" to "a key regulator of Bb formation" (line 230).

12) *Line 284: "suggests that the Bb replaces the.." consider instead "is important for recruiting this Bb regulator and possibly for Bb assembly."*

Response:
We revised the relevant text as suggested by the reviewer (lines 276-277).

13) *Line 286: "it thus sets a frame" . Consider instead "accordingly would set the foundation for.."*

Response:
W revised the relevant text as suggested by the reviewer (line 281).

Reviewer 2 Advance Summary and Potential Significance to Field:
Kikuchi et al have done a terrific job at reshaping the manuscript to a more comprehensive one by presenting in a clearer way the sex differences during germ cell ontology in medaka. The focus of the paper is now understandable. My concerns have been addressed even if the bibliography has been directed toward specific papers and my curiosity still want to see dazl mRNA localization from the MT dome formation to the Balbiani body as dazl is required for the persistence of the dome. Overall, the findings and the manuscript are of great interest for the field.

<u>Fourth decision letter</u>

MS ID#: DEVELOP/2023/201840

MS TITLE: Sexually dimorphic dynamics of the microtubule network in medaka (*Oryzias latipes*) germ cells

AUTHORS: Mariko Kikuchi, Miyo Yoshimoto, Tokiro Ishikawa, Yuto Kanda, Kazutoshi Mori, Toshiya Nishimura, and Minoru Tanaka

ARTICLE TYPE: Research Article

I am happy to tell you that your manuscript has been accepted for publication in Development, pending our standard ethics checks.

© 2024. Published by The Company of Biologists under the terms of the Creative Commons Attribution License (https://creativecommons.org/licenses/by/4.0/).
